# Genomic targets and selective inhibition of DNA methyltransferase isoforms

**DOI:** 10.1186/s13148-022-01325-4

**Published:** 2022-08-20

**Authors:** Chanachai Sae-Lee, Timothy M. Barrow, Elena Colicino, Si Ho Choi, Yoana Rabanal-Ruiz, Daniel Green, Viktor I. Korolchuk, John C. Mathers, Hyang-Min Byun

**Affiliations:** 1 Human Nutrition Research Centre, Centre for Healthier Lives, Population Health Sciences Institute, Newcastle University, Newcastle upon Tyne, UK; 2 Research Division, Faculty of Medicine, Siriraj Hospital, Mahidol University, Bangkok, Thailand; 3 Faculty of Health Sciences and Wellbeing, University of Sunderland, Sunderland, UK; 4 Department of Environmental Medicine and Public Health, Icahn School of Medicine at Mount Sinai, New York City, USA; 5 Research Center, Dongnam Institute of Radiological and Medical Sciences, Busan, 46033 Republic of Korea; 6 Biosciences Institute, Faculty of Medical Sciences, Newcastle University, Newcastle upon Tyne, UK; 7 Department of Medical Sciences, Faculty of Medicine, University of Castilla-La Mancha, Ciudad Real, Spain; 8 Institute of Life Course and Medical Sciences, Faculty of Health and Life Sciences, University of Liverpool, Liverpool, UK

**Keywords:** DNA methyltransferases, DNMT isoforms, Repetitive elements, Dietary constituents, Epigenetic clock, Epigenome, DNA methylation

## Abstract

**Background:**

DNA methylation in the human genome is established and maintained by DNA methyltransferases (DNMTs). DNMT isoforms show differential expression by cell lineage and during development, but much remains to be elucidated about their shared and unique genomic targets.

**Results:**

We examined changes in the epigenome following overexpression of 13 DNMT isoforms in HEK293T cells. We observed increased methylation (Δ*β* > 0.2) at 43,405 CpG sites, with expression of DNMT3A2, DNMTΔ3B4 and DNMTΔ3B2 associated with the greatest impact. De novo methylation occurred primarily within open sea regions and at loci with intermediate methylation levels (*β*: 0.2–0.6). 53% of differentially methylated loci showed specificity towards a single DNMT subfamily, primarily DNMTΔ3B and DNMT3A and 39% towards a single isoform. These loci were significantly enriched for pathways related to neuronal development (DNMTΔ3B4), calcium homeostasis (DNMTΔ3B3) and ion transport (DNMT3L). Repetitive elements did not display differential sensitivity to overexpressed DNMTs, but hypermethylation of *Alu* elements was associated with their evolutionary age following overexpression of DNMT3A2, DNMT3B1, DNMT3B2 and DNMT3L. Differential methylation (Δ*β* > 0.1) was observed at 121 of the 353 loci associated with the Horvath ‘epigenetic clock’ model of ageing, with 51 showing isoform specificity, and was associated with reduction of epigenetic age by 5–15 years following overexpression of seven isoforms. Finally, we demonstrate the potential for dietary constituents to modify epigenetic marks through isoform-specific inhibition of methylation activity.

**Conclusions:**

Our results provide insight into regions of the genome methylated uniquely by specific DNMT isoforms and demonstrate the potential for dietary intervention to modify the epigenome.

**Supplementary Information:**

The online version contains supplementary material available at 10.1186/s13148-022-01325-4.

## Background

DNA methylation within the human genome is established through the transfer of methyl groups from *S*-adenosylmethionine to the 5′ carbon of cytosine bases at CpG sites by DNA methyltransferases (DNMTs). This epigenetic modification, in tandem with histone modifications, serves to modulate accessibility of the DNA to transcription factors and thereby regulate gene expression, with critical roles in development and in cellular differentiation. In mammals, there are four DNMTs, of which three are catalytically active (DNMT1, DNMT3A and DNMT3B), while DNMT3L is not. Typically, these enzymes have N-terminal regulatory domains and C-terminal catalytic domains [1].

DNMT1 is a multi-modular protein comprising 1,620 amino acids, with a target recognition domain (TRD) and methyltransferase domain at its C-terminus [2], that has a primary role in maintenance of methylation patterns within the genome following cell division by recognition of hemimethylated DNA [3]. In contrast, DNMT3A and DNMT3B are involved in de novo methylation and are essential for establishment of global DNA methylation patterns [4, 5]. These show high homology, with conservation of the proline-tryptophan-tryptophan-proline (PWWP), C-terminal catalytic and cysteine-rich PHD zinc finger domains. Both isoforms are highly expressed in embryonic tissues but show reduced expression in differentiated somatic cells [4, 6]. The *DNMT3A* gene encodes two known isoforms, DNMT3A1 (~ 130 kDa) and DNMT3A2 (100 kDa), with the latter transcribed from an alternative promoter within intron 6 and lacking the N-terminal region [6]. DNMT3A2 is the predominant isoform in embryonic stem cells and shows greater association with euchromatin [6]. The *DNMT3B* gene encodes multiple isoforms which fall within two subfamilies: DNMT3B and DNMTΔ3B [7–10]. Members of the DNMT3B subfamily show tissue-specific expression patterns. DNMT3B1 and DNMT3B2 are catalytically active, while DNMT3B3, DNMT3B4, and DNMT3B5 are not [8]. Members of the DNMTΔ3B subfamily are derived from a promoter upstream of exon 5, resulting in truncated proteins lacking 200 amino acids at the N-terminal region. There are seven isoforms within this subfamily; DNMTΔ3B1-DNMTΔ3B4 are catalytically active while DNMTΔ3B5-7 lack the catalytic domain [11]. In addition to these catalytically active DNMTs, the human *DNMT3L* gene encodes a 387 amino acid protein containing a cystine-rich region with a novel-type zinc finger domain that is expressed only in embryonic stem cells and germ cells, where it regulates the expression of repetitive elements and imprinted genes [12–14]. DNMT3L enhances de novo methylation by catalytically active DNMT3A and DNMT3B isoforms [15, 16], e.g. through interaction with histone H3 tails to recruit DNMT3A2 [17].

Disruption of normal DNA methylation patterns is observed frequently in multiple human diseases, most notably cancers. Mutations within the *DNMT3A* gene are common in acute myeloid leukaemia [18] and are associated with global loss of methylation [19]. This has stimulated research on different approaches for inhibiting DNMT activity to restore normal DNA methylation patterns. Nucleoside analogues of cytosine such as azacytidine and decitabine have been approved for the treatment of leukaemias and lymphomas [20] but are cytotoxic, mutagenic and show lack of specificity. Non-nucleoside DNMT inhibitors may offer improved specificity and toxicity, with polyphenolic compounds gaining particular interest due to their anti-carcinogenic, anti-oxidative, and anti-inflammatory activities [21, 22], and with studies having demonstrated binding to the catalytic site of DNMTs in cancer chemoprevention [23].

The specificity and unique roles of DNMT3A and DNMT3B remain somewhat unclear. Studies to date have provided evidence for differential roles by cell type and stage of development [24, 25], with DNMT3B de novo methylation activity strongest during in embryonic development [26]. However, differences in their genomic targets have proven harder to establish. They appear to share many common targets but also show some distinct properties, including their relative activity at repetitive DNA sequences such as satellite repeats [27], with DNMT3B showing greater activity at centromeric DNA [4]. DNMT3A and DNMT3B appear to differ in their preferences according to DNA sequences flanking CpG sites [28] and by position of DNA in relation to nucleosomes [29], and there are known differences in non-CpG methylation [30]. However, the isoforms encoded by the *DNMT3A* and *DNMT3B* genes demonstrate distinct properties [31]. Early work in the field examined isoform-specific differences in methylation following overexpression, but using the limited Illumina Infinium GoldenGate methylation microarray that interrogates fewer than 2000 CpG sites across the genome [32]. To provide a more comprehensive examination of DNMT isoform specificity across the genome, here we performed epigenome-wide analysis of DNA methylation following overexpression of 13 DNMT isoforms. Further, we examined the impact of these isoforms upon epigenetic models of ageing and the potential use of dietary constituents to achieve isoform-specific inhibition of methylation activity.

## Results

### Generation of stable isoform-overexpressing cell lines

**Fig. 1:**
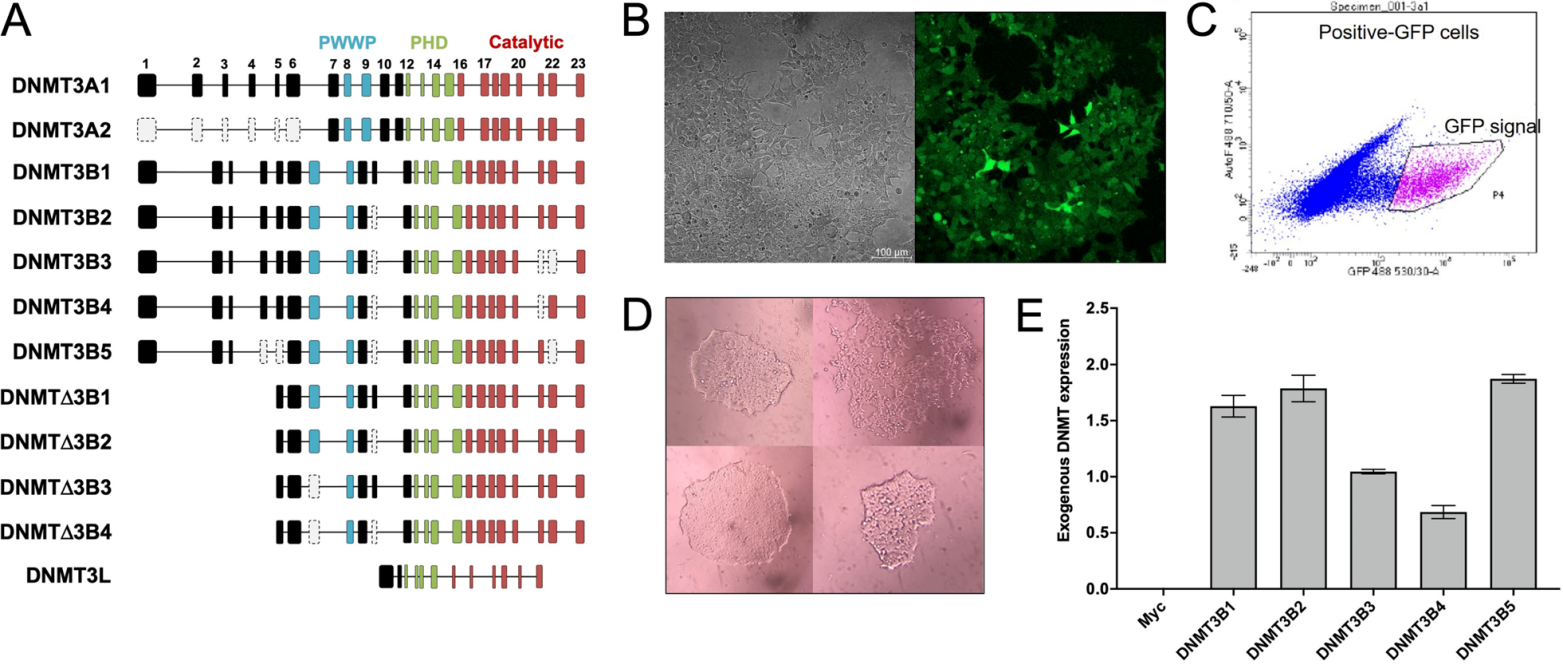
Construction of DNMT overexpressing cell lines. **A** Diagrammatic representation of isoforms from the DNMT3A, DNMT3B, DNMTΔ3B and DNMT3L subfamilies. Exons are indicated, with missing or excluded exons in grey. Also indicated are the proline-tryptophan-tryptophan-proline (PWWP) domain (blue), cysteine-rich PHD zinc finger domain (green), and C-terminal catalytic domain (red). DNMT1 is not illustrated due to its highly divergent N-terminal region. **B** Bright field and fluorescence microscope images of HEK293T cells transduced with the DNMT3L construct, expressing GFP. **C** Selection of GFP-positive (pink) transduced cells by FACS. **D** Morphology of single-cell colonies grown in cells transduced to overexpress DNMTΔ3B4. **E** Exogenous expression of DNMT3B1-5 in transduced cells, relative to *PPIA* and *GAPDH*. Data are expressed as means ± SD

HEK293T cells were transduced using plasmid constructs containing gene sequences for 13 DNMT isoforms: *DNMT1*; *DNMT3A1*; *DNMT3A2*; *DNMT3B1*: *DNMT3B2*; *DNMT3B3*; *DNMT3B4*; *DNMT3B5*; *DNMTΔ3B1*; *DNMTΔ3B2*; *DNMTΔ3B3*; *DNMTΔ3B4*; and *DNMT3L*. Molecular structures of the isoforms are provided in Fig. 1A, with comparison of their amino acid sequences in Additional file 1: Table S1. Transduced cells expressing the isoforms were identified via GFP expression (Fig. 1B) and cultured for 17 days before isolation by FACS (Fig. 1C), with single-cell clones subsequently grown in 96-well plates (Fig. 1D). DNMT isoform overexpression was confirmed by qPCR (Fig. 1E). The HEK293T cell line displays low endogenous expression of *DNMT3A1*, *DNMT3B1-5*, *DNMTΔ3B1-4* and *DNMT3L*, and high expression of *DNMT3A2* (Additional file 1: Figure S1).

### Differential methylation following DNMT isoform overexpression

**Fig. 2:**
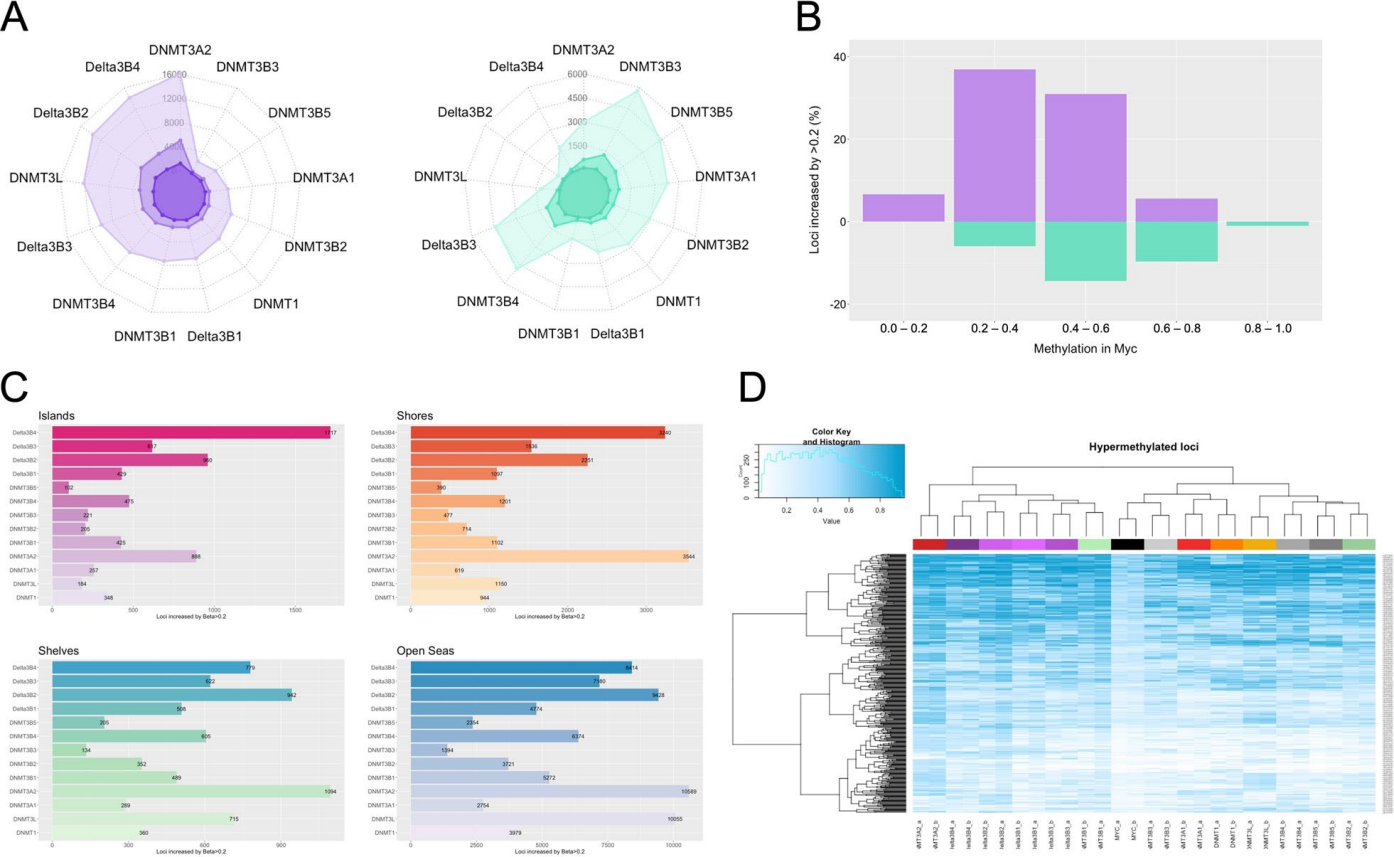
Characterisation of changes in DNA methylation in response to DNMT isoform overexpression. **A** Number of CpG sites displaying gains (left, purple) and losses (right, green) of Δ*β* > 0.4 (dark purple/green), Δ*β* > 0.3 (medium purple/green) and Δ*β* > 0.2 (light purple/green) by DNMT isoform. **B** Frequency of gains of Δ*β* > 0.2 (purple) and losses of Δ*β* < − 0.2 (green) across all isoform-overexpressing cell lines by methylation levels in control cells. **C** Number of CpG sites displaying gains of Δ*β* > 0.2 mapping to CpG islands, shores, shelves and open seas by DNMT isoform. **D** Heatmap displaying methylation at loci displaying gains of methylation of Δβ > 0.3 in response to expression of one or more isoforms

Epigenome-wide analysis of DNA methylation was performed for the 13 isoform-overexpressing cell lines and control cells using the Illumina MethylationEPIC microarray. Following normalisation and quality control processes, DNA methylation at 770,733 CpG sites was examined. A total of 43,405 different CpG sites displayed increases in DNA methylation (Δ*β* > 0.2) in response to overexpression of one or more DNMT isoforms, representing < 6% of all interrogated loci. Overexpression of DNMT3A2 (16,115 CpG sites), DNMTΔ3B4 (14,150 CpG sites) and DNMTΔ3B2 (13,581 CpG sites) altered methylation at the greatest number of CpG sites, with DNMT3B3 (2226 CpG sites) and DNMT3B5 (3,051 CpG sites) affecting the fewest sites (Fig. 2A, left; Additional file 1: Table S2). Interestingly, overexpression also resulted in losses of methylation (Δ*β* < − 0.2), although less frequently. The catalytically inactive DNMT3B3 (5759 CpG sites), DNMT3B4 (4808 CpG sites) and DNMT3B5 (4311 CpG sites) isoforms, in addition to DNMTΔ3B3 (4391 CpG sites), were associated with losses at the greatest number of loci (Fig. 2A, right). The impacts upon mean global methylation levels were subtle. Overexpression of DNMTΔ3B2 and DNMT3A2 was associated with increases of 0.03 and 0.02 in global mean methylation (β), respectively, while overexpression of DNMT3B3 was associated with a 0.01 loss in global mean methylation.

Most changes in DNA methylation did not occur at unmethylated (*β* < 0.2) or highly methylated (*β* > 0.8) loci, but rather at loci with intermediate values. Methylation gains were observed most frequently at loci with methylation levels (*β*) of between 0.2 and 0.6 in control cells, while losses were observed at those with levels of 0.4–0.8 (Fig. 2B). Most gains in methylation occurred in open sea regions, with only 1.5–12.1% mapping to CpG islands (Fig. 2C; Additional file 1: Figure S2).

Hierarchical clustering revealed correlated methylation patterns between cells overexpressing the four delta isoforms (DNMTΔ3B1 − DNMTΔ3B4), but characteristically different methylation patterns between DNMT3A1 and DNMT3A2 and between DNMT3B1 and DNMT3B2 (Fig. 2D). Together with the total number of differentially methylated loci among the DNMT3A and catalytically active DNMT3B isoforms, this indicates specificity of even the most closely related enzymes.

### Specificity of differentially methylated sites to isoforms

**Fig. 3:**
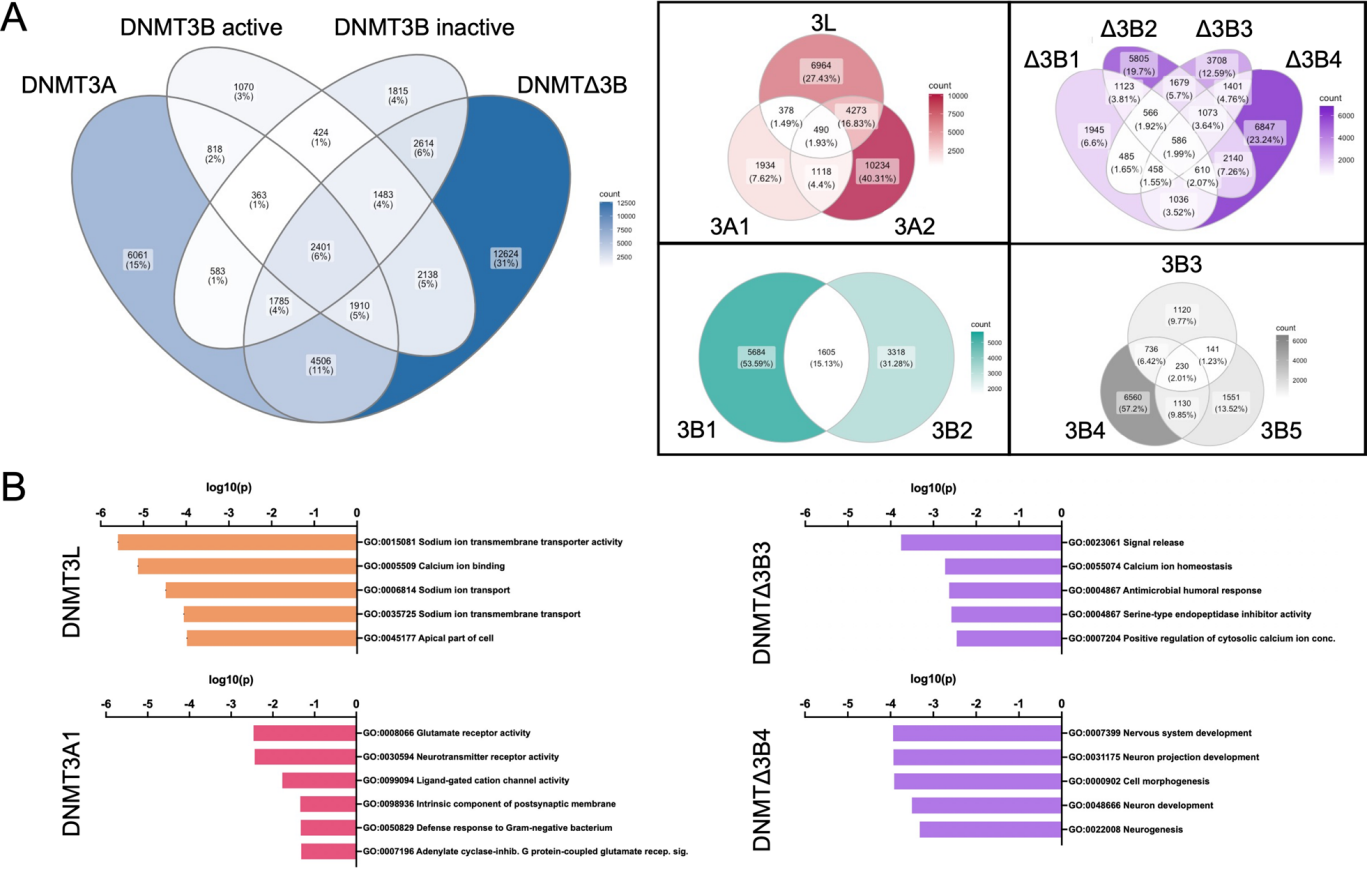
Specificity of hypermethylated loci to DNMT subfamilies and isoforms. **A** Venn diagram illustrating CpG sites showing gains of methylation of Δ*β* > 0.2 by DNMT subfamily (blue) and by isoform within the DNMT3A (red), DNMT3B catalytically active (green), DNMT3B catalytically inactive (grey) and DNMTΔ3B (purple) subfamilies. **B** GO pathway analysis for CpG sites displaying gains of Δ*β* > 0.2 uniquely in response to overexpression of DNMT3L, DNMT3A1, DNMTΔ3B3 and DNMTΔ3B4

To examine the isoform specificity of these de novo methylation events, we first compared the DNMT3A (DNMT3A1 and DNMT3A2), catalytically active DNMT3B (DNMT3B1 and DNMT3B2), catalytically inactive DNMT3B (DNMT3B3, DNMT3B4 and DNMT3B5) and delta (DNMTΔ3B1, DNMTΔ3B2, DNMTΔ3B3 and DNMTΔ3B4) subfamilies. Of the 40,595 loci displaying gains in methylation of > 0.2 among these groups, 53% demonstrated specificity to a single subfamily (Fig. 3A). This specificity was most pronounced for the delta (31%) and DNMT3A (15%) subfamilies, with only 3% and 4% specific to the catalytically active and inactive DNMT3B subfamilies, respectively. In contrast, 6% of loci showed high promiscuity with increased methylation with each of the four subfamilies, and a further 23% displayed changes in two or three of the catalytically active subfamilies.

Isoform specificity was also apparent within subfamilies. This was most evident amongst the delta subfamily, for which 62% of hypermethylated loci were specific to a single isoform (Fig. 3A). Among DNMT3As, 40% of loci were specific to DNMT3A2 in comparison with only 8% specific to DNMT3A1, while for the catalytically active DNMT3B isoforms 54% of loci were specific to DNMT3B1, 31% were specific to DNMT3B2, and only 15% were shared.

Overall, a total of 17,124 loci were hypermethylated in response to overexpression of a single specific DNMT isoform (39.4% of all hypermethylated loci). DNMT3A2 and DNMTΔ3B4 had the greatest number of unique loci (4294 and 3487, respectively), with only 96 loci unique to DNMT3B3 and 176 unique to DNMT3B5. Verification by pyrosequencing confirmed the accuracy of our observations by microarray of increased methylation at selected target loci (Additional file 1: Figure S3).

### Functional enrichment of target loci

Pathway analysis revealed enrichment of hypermethylated loci within cellular pathways associated with neuronal function and immune response. Unbiased GO analysis revealed enrichment of 226 pathways, of which 65 relate specifically to neuronal function and voltage-gated ion channels, with components of plasma membranes also in abundance. The majority (134) were specific to overexpression of a single isoform, of which 90 were associated with members of the DNMTΔ3B subfamily. Only nine pathways were unique to DNMT3B, six to DNMT3A, and none to DNMT1. The isoforms with most unique pathways were DNMTΔ3B4 (51), DNMTΔ3B3 (38) and DNMT3L (29), which displayed strongest associations with neuronal development, calcium homeostasis, and sodium and calcium transport, respectively (Fig. 3B). A further six pathways were specific to DNMT3A2, with five of these related to glutamate/neurotransmitter activity. Furthermore, the most enriched but non-unique pathways related to DNMT3A1 overexpression were also associated with synaptic signalling.

By KEGG analysis, loci associated with neuroactive ligand-receptor interaction were significantly enriched with overexpression of DNMT3A1, DNMT3B2, DNMT3B4, DNMTΔ3B3 and DNMTΔ3B4 (Additional file 1: Table S3). Loci associated with nicotine addiction were enriched following overexpression of DNMT1 and DNMTΔ3B3. Cytokine-cytokine interaction was enriched with overexpression of DNMT3L and DNMTΔ3B3, and viral protein interaction with cytokine and cytokine receptor were enriched with DNMT1 and DNMT3L.

### Changes within repetitive elements

**Fig. 4:**
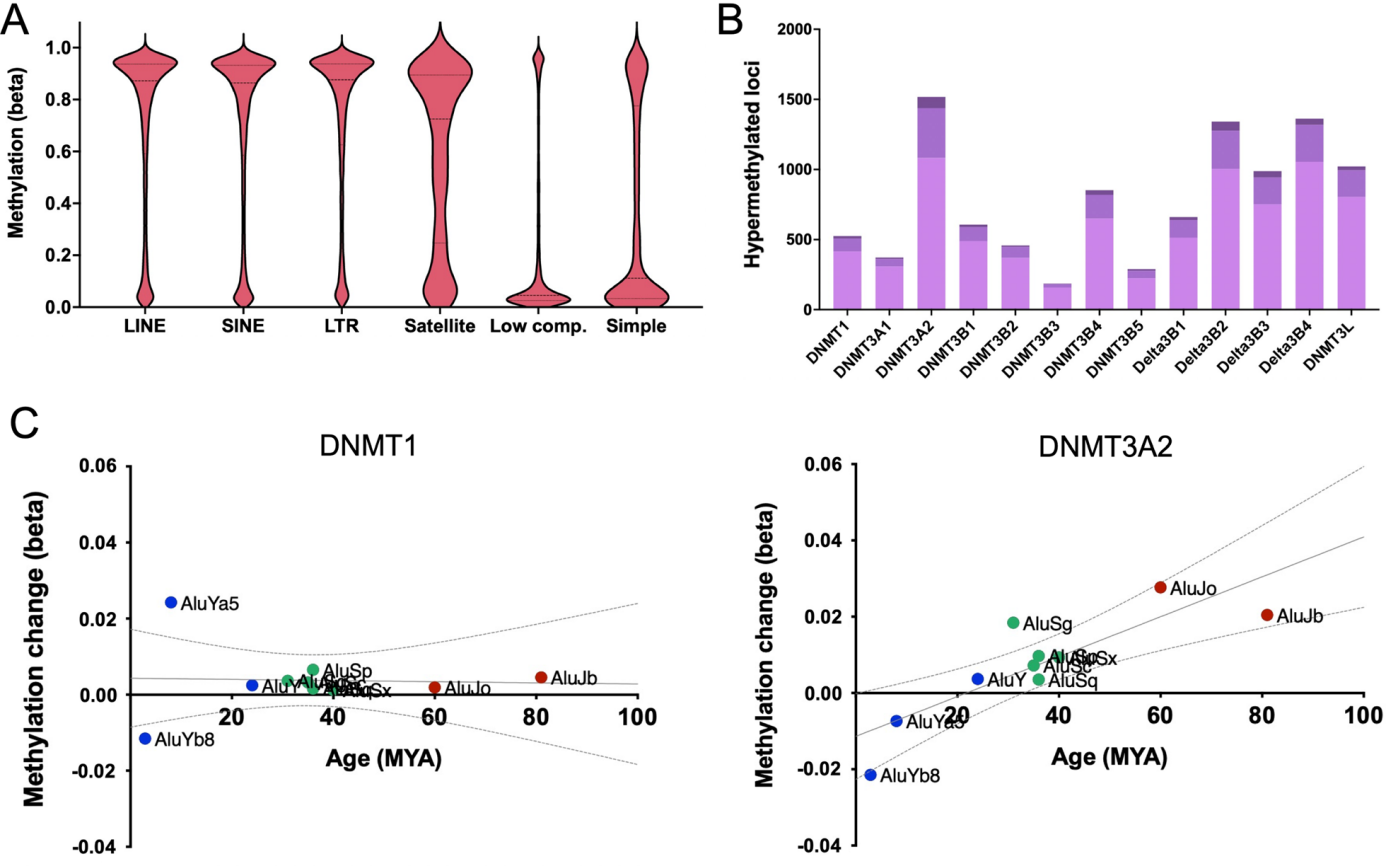
Analysis of repetitive elements. **A** DNA methylation at loci mapping to LINES, SINES, long terminal repeats (LTR), satellite DNA, regions of low complexity, and simple repeats. **B** Gains of methylation of Δ*β* > 0.2 (light purple), Δ*β* > 0.3 (medium purple) and Δ*β* > 0.4 (dark purple) at loci mapping to repetitive elements in response to DNMT isoform overexpression. **C** Correlation between changes in DNA methylation (Δ*β*) at *Alu* subfamilies by their time since integration within the genome (million years ago, MYA)

More than half of the human genome is comprised of repetitive elements. We examined the impact of DNMT isoform overexpression on DNA methylation at these regions, with particular focus on long and short interspersed nuclear elements (LINEs and SINEs), long terminal repeats (LTRs), and satellite DNA. These four groups of repetitive elements displayed very high levels of methylation in control cells, while regions of low complexity and simple repetitive sequences were predominantly unmethylated (Fig. 4A). Similar to other regions of the genome, the overexpression of DNMT3A2, DNMTΔ3B2 and DNMTΔ3B4 resulted in gains of methylation (Δ*β* > 0.2) at the greatest number of loci, with few gains observed with DNMT3B3 and DNMT3B5 (Fig. 4B). LINEs, SINEs, LTRs and satellite DNA did not display differential sensitivity to DNMT isoform overexpression, with 4.2–6.5% of CpG sites mapping to each element type displaying a gain in methylation of > 0.2 and 0.2–0.5% displaying gains of > 0.4%. These results again suggest a comparatively moderate impact of DNMT overexpression upon the epigenome. Interestingly, however, for four DNMT isoforms the changes in methylation at *Alu* elements were associated with their evolutionary age: DNMT3A2 (*r*^2^ = 0.71; pFDR = 0.03) (Fig. 4C, right); DNMT3B1 (*r*^2^ = 0.58; pFDR = 0.04); DNMT3B2 (*r*^2^ = 0.60; pFDR = 0.04); and DNMT3L (*r*^2^ = 0.57; pFDR = 0.04). Methylation changes associated with overexpression of other isoforms, such as DNMT1, showed no such relationship (Fig. 4C, left). In contrast to other reports [4, 27], satellite DNA was not specifically methylated by DNMT3B.

### Analysis of chromosome X

The X chromosome displays unique epigenetic regulation in comparison with autosomes, with silencing of one X chromosome in females. To determine the impact of DNMT isoform overexpression upon X-inactivation, we examined DNA methylation at the 16,700 CpG sites mapping to this chromosome. In control cells, most loci display intermediate methylation levels, with reduced frequency of unmethylated (*β* < 0.2) or fully methylated (*β* > 0.8) loci (Additional file 1: Figure S4). In transduced cells overexpressing DNMT isoforms, we identified 1074 loci displaying increased methylation (Δ*β* > 0.2). Similar to their epigenome-wide impact, overexpression of DNMTΔ3B4 (463 loci), DNMTΔ3B3 (323), DNMTΔ3B2 (300), and DNMT3A2 (297) induced gains of methylation at the greatest number of loci. Larger gains (Δ*β* > 0.4) were rare and were observed at only 51 loci across the chromosome. Gains were significantly enriched at open seas (Fisher’s exact test, *p* < 0.0001) and depleted at CpG islands (*p* < 0.0001). At the *XIST* locus, with a key role in maintenance of X-inactivation, two loci displayed changes of Δ*β* > 0.2. One of these, cg03862022, showed increased methylation following overexpression of DNMT1, DNMT3A1, DNMT3A2, DNMT3B4, DNMTΔ3B2, DNMTΔ3B3 and DNMT3L, and maps to a binding site for the TAF1, TBP and YY1 transcriptional regulators. The other, cg00378994, showed increased methylation with overexpression of DNMTΔ3B4 and maps to a RAD21 and CTCF binding site.

### Loci associated with ‘epigenetic age’

Over the past decade, there has been great interest in epigenetic models of ageing. To examine the potential influence of DNMT expression and activity upon epigenetic estimates of biological age, we examined the 353 CpG sites that comprise the Horvath ‘epigenetic clock’ model [33]. While 19 of these are missing from the Illumina EPIC microarray, this does not have a significant effect on estimates of epigenetic age [34]. DNMT overexpression had widespread impact on these sites, with gains of methylation of Δ*β* > 0.1 at 83 loci (25%) and losses of Δ*β* < − 0.1 at 38 loci (11%). Approximately half of these loci displayed isoform specificity: 44 of the sites with increased methylation and 17 with decreased methylation displayed changes in response to overexpression of only one or two specific DNMT isoforms. In contrast, 30 loci showed a high degree of promiscuity, with gains and losses occurring in response to the overexpression of five or more different isoforms. The most frequent gains in methylation were following overexpression of DNMTΔ3B4 (50 loci), DNMT3A2 (35 loci) and DNMT3B1 (24 loci), while losses were most frequently observed following overexpression of the three catalytically inactive DNMT3B isoforms (14–16 loci). Larger gains of Δ*β* > 0.2 were observed at 30 of the loci, and gains of Δ*β* > 0.4 at four. These were observed primarily following overexpression of DNMT3A2 and DNMTΔ3B4, which led to such changes at 19 and 15 of these loci, respectively; no other isoform was associated with large gains at more than 7 loci.

**Fig. 5:**
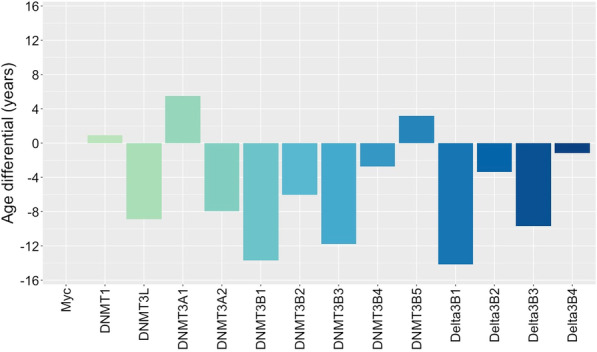
Impact of DNMT isoform overexpression of epigenetic ageing. Changes in estimates of epigenetic age (‘Age differential’, years) in cell lines overexpressing DNMT isoforms, calculated by the Horvath ‘epigenetic clock’ model of ageing

To determine the impact of these changes, we calculated the epigenetic age for control cells and isoform-overexpressing cells. While the ‘age’ of cultured cells is unlikely to reflect the chronological age of the donor from which they were isolated, having been impacted by immortalisation and proliferation, comparison of overexpressing and control cells enables the impact of DNMT activity upon epigenetic age to be assessed directly. We observed a significant reduction in epigenetic age in response to overexpression of most DNMT isoforms. Interestingly, however, the magnitude of change in epigenetic age was not directly correlated with the number of loci displaying gains or losses in methylation. Overexpression of DNMT3L, DNMT3A2, DNMT3B1, DNMT3B2, DNMT3B3, DNMTΔ3B1 and DNMTΔ3B3 were all associated with a reduction in epigenetic age of 5–15 years (Fig. 5). Increased epigenetic age was observed only with DNMT3A1 (5.5 years) and DNMT3B5 (3.2 years).

### Isoform-specific inhibition of DNMT activity by caffeic acid

DNMT activity is modifiable through dietary factors that can act as methyl donors or through direct enzymatic inhibition. Computer modelling suggests that polyphenolic compounds, such as those found in teas and coffees, can bind within the active site DNMTs such as DNMT1 and DNMT3A to act as competitive inhibitors [35–37]. This is supported by in vitro evidence suggesting that they can directly inhibit DNMT1 activity [38] and modify epigenetic patterns in cell lines [36]. These can show differential inhibition of different DNMTs, with epigallocatechin-3-O-gallate (EGCG) a more potent inhibitor of DNMT1 than DNMT3A [39].

**Fig. 6:**
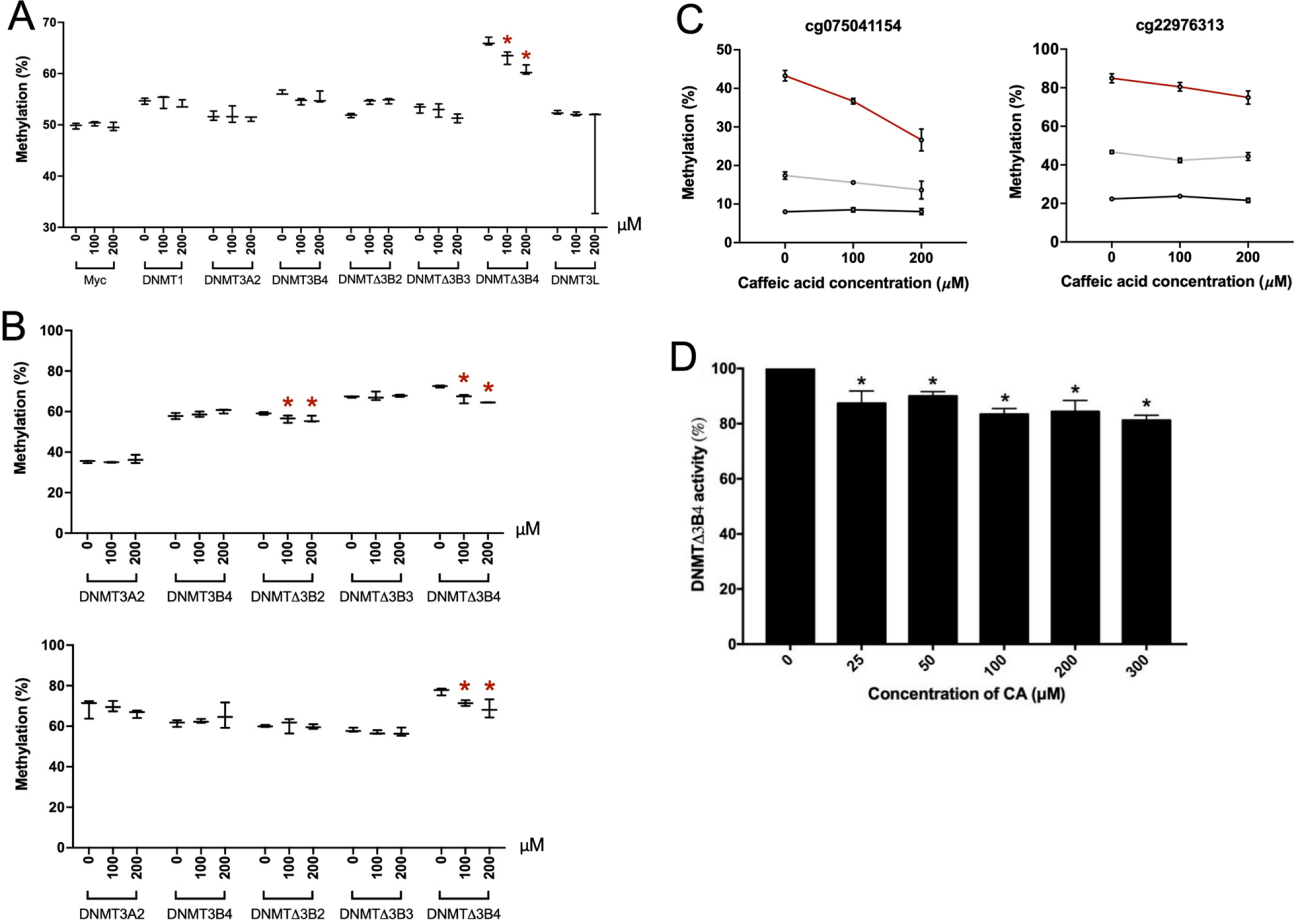
Inhibition of DNMTΔ3B4 by caffeic acid. **A** Changes in global methylation (%), measured by LINE-1 assay, in response to exposure to 0, 100 and 200 µM caffeic acid in cell lines expressing seven DNMT isoforms. **B** Changes in methylation at cg25843713 (top) and cg04458645 (bottom) in response to caffeic acid exposure. **C** Changes in methylation at two loci uniquely methylated by DNMTΔ3B4 (cg07504154, left; cg22976313, right) in response to caffeic acid exposure. Methylation in control cells (black) and those overexpressing DNMT3L (grey) and DNMTΔ3B4 (red) are displayed. **D** Methylase activity of DNMTΔ3B4 in the presence of 0–300 µM caffeic acid. Data are presented as means ± SD

Caffeic acid is a polyphenolic compound found in coffee and in some fruits, herbs and spices that has been shown to inhibit methylation of DNA [40], with its phenyl ester a potent inhibitor of DNMT1 [35]. We therefore investigated the potential of caffeic acid to selectively inhibit DNMT isoforms. We treated cell lines overexpressing DNMT1, DNMT3L, DNMT3A2, DNMT3B4, DNMTΔ3B2, DNMTΔ3B3 and DNMTΔ3B4 with caffeic acid for 48 h before analysing global DNA methylation levels by LINE-1 assay. Our results revealed isoform-specific inhibition of enzymatic activity. Global DNA methylation levels were moderately but significantly reduced in DNMTΔ3B4 overexpressing cells, but no significant effect was observed in the other cell lines (Fig. 6A). To examine locus-specific changes, we analysed two CpG sites, cg25843713 and cg04458645, which we had identified previously as being hypermethylated in each of these seven overexpressing cell lines. Both loci showed reduced methylation in the DNMTΔ3B4 overexpressing cell line, while cg25843713 additionally showed reduced methylation in DNMTΔ3B2 overexpressing cells (Fig. 6B). We then examined the impact of caffeic acid on loci that we had identified previously as selectively methylated by DNMTΔ3B4: cg22976313 and cg07504154. Treatment with caffeic acid again resulted in a moderate, but statistically significant, reduction in methylation at both loci that was specific to DNMTΔ3B4 overexpressing cells. Treatment with 200 μM caffeic acid resulted in a 17% reduction in methylation at cg075041154 and 10% at cg22976313, while methylation was unaffected in control cells and in those overexpressing DNMT3L (Fig. 6C).

Together, this demonstrates the potential for caffeic acid to selectively inhibit the DNMTΔ3B4 isoform to modulate epigenetic marks. To investigate the mechanism behind this effect, we analysed the enzymatic activity of purified DNMTΔ3B4 through an in vitro colorimetric assay in the presence of increasing doses of caffeic acid. In line with our observation of moderate changes in DNA methylation, we similarly observed a moderate but significant reduction in enzymatic activity with doses of 25, 50, 100, 200 and 300 μM (Fig. 6D), suggesting modification of epigenetic patterns through direct inhibition of DNMT activity.

## Discussion

The DNMT family plays a crucial role in the regulation of gene expression via DNA methylation. The heritability of these epigenetic marks is ensured through the activity of DNMT1, while de novo methylation is conferred by DNMT3A and DNMT3B. While much is known about the differential expression of DNMT3A and DNMT3B by cell type and differentiation status [24, 25, 41], their shared and unique genomic targets have not been well characterised and understood. Most studies have sought to characterise their distinct roles through knockdown or inactivation [26, 42], and from the study of diseases such as acute myeloid leukaemia in which DNMT3A is frequently mutated. However, many studies have focussed on the roles of particular isoforms in a specific cell type or point in development (very commonly embryonic stem cells) when it is known that DNMT3A and DNMT3B isoforms show lineage-specific expression patterns and differences in their recruitment and regulation. Here, we comprehensively characterised changes in DNA methylation in a differentiated cell line in response to overexpression of 13 different DNMT isoforms to bring insight into their unique cellular roles. Our results demonstrate subtle but potentially impactful changes within the epigenome in response to increased methyltransferase activity of different isoforms.

We observed locus specificity at two levels. Firstly, more than half of the loci showing gains of methylation did so in response to overexpression of only one DNMT subfamily (DNMT1, DNMT3A, DNMT3B, DNMTΔ3B, or DNMT3L). Secondly, we have demonstrated that there is substantial isoform specificity within these families, with little overlap between DNMT3A1 and DNMT3A2, DNMT3B1 and DNMT3B2, and DNMTΔ3B1-4. This may initially appear in contrast to observations elsewhere of very high levels of redundancy among the genomic targets of DNMT3A and DNMT3B [42] and of high redundancy between isoforms within families [32]. However, our findings do not exclude the possibility that there is a high degree of redundancy between isoforms. Our approach comprised overexpression of specific isoforms in cell lines in which other DNMTs are also expressed, and subsequently we observed comparatively modest changes within the epigenome, with ~ 6% of loci showing gains of methylation. Those regions of the genome already showing high levels of methylation may be targeted by multiple DNMT families and/or isoforms, which is more likely to be revealed through knockdown studies. Indeed, in mouse knockout models it has been reported that 96% of all differentially methylated regions retained their methylation unless both DNMT3A and DNMT3B were lost [42]. While many regions may be redundantly targeted, our study has revealed loci within the human genome that are preferentially methylated by specific isoforms. It should be noted that there are likely to be further loci displaying isoform specificity, but which are challenging to identify due to selection pressure against methylation at these regions, such as those regulating expression of housekeeping genes. Indeed, an inherent challenge in studies of DNMT activity is their critical role in the cell, demonstrated by the embryonic [42] and post-natal [4] lethality of loss of their activity. It is likely that these loci will need to be identified through rational computational approaches, rather than direct experimental evidence.

Overexpression of DNMT3A2 and the DNMTΔ3B1-4 isoforms resulted in the most widespread gains of methylation, with DNMT1 and the catalytically inactive DNMT3B isoforms resulting in comparatively few gains. This is in agreement with the observation of Choi et al.[32] that hypermethylation is associated mostly strongly with the DNMT3B subfamily. However, while they reported that most changes occurred in unmethylated regions of the genome, we observed that gains of methylation rarely occurred in such areas and instead were more common in those with intermediate methylation levels. This discrepancy may be technical rather than biological, with the Illumina EPIC methylation microarray offering a more comprehensive analysis of the epigenome, both in terms of the number of CpG sites (~ 850,000) and broader coverage of functional elements and regions, in comparison with the GoldenGate microarray (1536 CpG sites). Furthermore, while GoldenGate microarray probes are enriched for CpG islands, we observed most gains of methylation occur in open seas. Indeed, our findings provide further evidence for DNMT isoforms showing differential specificity by CpG density, with DNMTΔ3B4 overexpression leading to a greater proportion of gains within CpG islands, while DNMT3L showed specificity for low-density open seas. Importantly, our results show that increased DNMT expression and activity is unlikely to ‘force’ methylation at novel loci, but rather may achieve more complete methylation of previously heterogenous regions. Subsequently, the broader impact of DNMT overexpression upon global methylation levels may be comparatively subtle and without resulting in substantial modulation of the epigenome.

Repetitive elements within the genome were not disproportionately affected by DNMT overexpression. Satellite DNA sequences are known to be sensitive to loss of DNMT3B expression [42, 43], but our findings do not indicate sensitivity to increased expression. However, among SINEs we observed that *Alu* elements showed differential sensitivity according to their evolutionary age, with older *Alu*J elements showing greater gains in methylation following overexpression of DNMT3A2, DNMT3B1, DNMT3B2 and DNMT3L. Similar differential sensitivity to changes in methylation by evolutionary age has been reported in response to environmental exposures [44] and in chronic lymphocytic leukaemia [45], but the mechanisms for this remain unclear. DNMT3B isoforms have stronger affinity for transposable elements than those from the DNMT3A subfamily, although this is more pronounced among LINEs than SINEs [26].

Expression of DNMT isoforms lacking a catalytical domain influence epigenomic patterns via interactions with active DNMTs. DNMT3L is a stimulator of de novo methylation by DNMT3A and DNMT3B [15, 16], particularly at retrotransposons [14], and we observed a high number of gains with DNMT3L overexpression, particularly within open sea regions. Other catalytically inactive isoforms have been demonstrated to both stimulate [46–48] and inhibit [31, 49] de novo methylation, underlining the complexity in understanding such processes. DNMT3B3 and DNMT3B4 are interact directly with DNMT3A2, DNMT3B1 and DNMT3B2 to inhibit de novo methylation by weakening their binding to DNA [31]. In line with this, we observed that overexpression of DNMT3B3 and DNMT3B5 resulted in more losses of methylation than gains, while DNMT3B4 was also led to loss of methylation at multiple loci. Interestingly, substantial losses of methylation were also observed with overexpression of catalytically active isoforms. Such negative correlations between DNMT expression and locus-specific methylation have been reported elsewhere [50], and we speculate that this may be result from alterations in the expression of other isoforms in response to induced overexpression. The inability to directly assay endogenous levels of each isoform following this overexpression, due to the absence of isoform-specific commercially available antibodies, is a limitation of our study. Furthermore, as we have not directly assessed the nuclear localisation and enzymatic function of the exogenous isoforms, we cannot exclude that the observed changes in DNA methylation could in part be through other unconsidered and non-specific effects.

Differentially methylated loci were enriched in pathways related to neuronal function, cellular signalling and transport across membranes. DNMT3A1 overexpression resulted in altered methylation of genes related to synaptic signalling and glutamate activity. The DNMT3A subfamily is expressed in young neurons and may induce tissue-specific methylation patterns [51], although studies elsewhere have reported that DNMT3A2 expression, but not that of DNMT3A1, is induced in hippocampal neurons by synaptic activity and is crucial for maintenance of cognitive abilities [52].

Our findings may help to bring insight into disease processes by understanding and predicting the potential impact of changes in DNMT isoform expression. For example, DNMT3A2 and DNMT3B2 show increased expression in prostate tumours, and overexpression of DNMT3B2 in primary prostate cells introduces methylation gains at loci known to be differentially methylated in prostate tumours [53]. Therefore, it is important to identify redundancy and specificity to different DNMT isoforms within the genome. Furthermore, our observation that DNMT overexpression causes large falls in estimates of epigenetic age warrants follow-up. In that context, we emphasise that our observations represent proof of principle in a single cell type only, but they suggest the intriguing possibility that disruption of normal DNMT expression and activity could impact upon biological ageing and, consequently, health.

Importantly, our study has also suggested that isoform-specific inhibitors may have potential in modifying epigenetic marks. While epigenetic therapies such as decitabine have been approved for clinical use, they are indiscriminate and wide-ranging in their effects upon the epigenome, and they are cytotoxic. Cas9 technology may offer a much more targeted approach to methylating or demethylating specific loci [54], but currently this is technically very challenging. Our proof-of-principle study demonstrated that caffeic acid (found in multiple plant foods) selectively inhibits the capacity of specific DNMT isoforms to modify DNA methylation at certain loci. As we have not directly compared the relative enzymatic activity of endogenous and (Myc-tagged) exogenous DNMTΔ3B4, it is possible that the level of inhibition may differ between native and recombinant forms of DNMTs. While we achieved modest inhibition of DNMTΔ3B4 in vitro, this was sufficient to reduce DNA methylation by 10% at loci specifically methylated by this isoform. The effects across the epigenome may be specific and therefore comparatively moderate, as caffeic acid-treated cell lines do not show significant loss of global methylation levels [55]. Since numerous dietary components are known or hypothesised to be inhibitors of DNMT activity, including curcumin, ECGC, theaflavin and vitamin C [56], our findings may provide support for dietary interventions to modify epigenetic patterns. This builds on our previous work which demonstrated that dietary supplementation with the methyl donors folic acid and vitamin B12 modulates epigenetic age [57]. As an example of its potential application, DNMT3A2 is a modulator of chronic inflammatory pain and therefore inhibiting its activity has been suggested as a means to reduce hypersensitivity in patients [58].

### Conclusions

Together, our results have provided further insight into the genomic specificity of DNMT isoforms, revealing a moderate effect of increased expression and activity. Intriguingly, such overexpression had marked effects on epigenetic models of ageing. Our results have also suggested that isoform-specific inhibitors, such as caffeic acid, could be utilised to modify epigenetic marks at loci showing isoform specificity.

## Methods

### Cell culture

HEK293T and DNMT-overexpressing cells were cultured in Dulbecco’s Modified Eagle’s Medium (DMEM) high glucose (Sigma, UK) supplemented with 10% FBS (Sigma, UK), and 2 mM glutamine, at 37 °C in a 5% CO_2_ humidified atmosphere.

### Generation of cell lines with stable overexpression of specific DNMT isoforms

pIRES puro3 plasmids containing genes encoding individual DNMT isoforms with an N-terminal Myc tag were sub-cloned into pLenti7.3/V5 DEST gateway vector. Expression of the DNMT isoform was under control of a CMV promoter. The vector also contains a gene encoding containing green fluorescent protein, under the control of an SV40 promoter, for selection of transduced cells. The pLenti7.3/V5 DEST gateway vector was modified to introduce the multiple cloning site (containing XbaI, NheI, ClaI, EcoRI, SwaI, PspOMI, and MluI sites). All plasmids were confirmed by sequencing. Lentiviral constructs were transduced into HEK293T cells in the presence of polybrene (Sigma, USA).

### Single-cell selection

Transduced cells were cultured for 17 days and sorted in a single drop into each well of a 96-well plate using FACS Fusion Sorter. GFP expression was assessed by fluorescence microscopy and clones were cultured until formation of single colonies.

### Analysis of exogenous expression by qRT-PCR

Exogenous expression of DNMT isoforms was assessed by qRT-PCR. Total RNA was extracted using the E.Z.N.A.® Total RNA kit I (OmegaBiotek, USA) according to the manufacturer’s instructions. Total RNA was quantified by NanoDrop™ 2000 (Thermo Scientific, USA). cDNA was generated from 1 μg of total RNA using the Omniscript RT kit (Qiagen, USA) and random hexamer primers, according to the manufacturer’s instructions. Gene expression was quantified using isoform-specific primers (Additional file 1: Table S4) and 2 × QuantiTect SYBR green PCR master mix (Qiagen, USA). Primer pairs were specific to *DNMT3A1*, *DNMT3A2*, *DNMT3B* (capturing *DNMT3B1-5*), DNMT∆3B1-2 (capturing *DNMT∆3B1 and DNMT∆3B2*), DNMT∆3B3-4 (capturing *DNMT∆3B3 and DNMT∆3B4*), DNMT1, and DNMT3L. Each set of primers was designed to amplify unique mRNA transcripts avoiding non-specific targets from other DNMT isoforms. A universal forward primer corresponding to the Myc sequence was used in conjunction with an isoform-specific reverse primer. To identify appropriate endogenous controls, we analysed expression of 11 housekeeping genes (*ACTB*, *B2M*, *GAPDH*, *GUSB*, *HPRT1*, *PPIA*, *RPL13A*, *RPLP0*, *RPS13*, *TFRC*, and 18sRNA) and selected the two showing most stable expression, *GAPDH* and *PPIA*, for normalisation based on geNorm analysis [59].

### DNA methylation analysis by Illumina Infinium methylation EPIC BeadChip

The Illumina Infinium Methylation EPIC BeadChip (EPIC array) was used to measure de novo DNA methylation for each individual DNMT cell. Bisulfite-converted DNA from biological duplicates of cell lines expressing each DNMT isoform was used for microarray-based analysis, performed by Eurofins Genomics. Each biologically duplicated DNMT-overexpressing cell was selected on the basis of exogenous *DNMT* expression*.* All IDAT files were imported and analysed in R Studio using the Bioconductor package. Probes with high-detection *p*-values (> 0.01) were removed. PreprocessNoob was utilised for normalisation, leaving 866,091 probes after this process, which were then adjusted for probe-type bias for Infinium I (type I) and Infinium II (type II) probes [60]. Cross-reactive probes (43,254 loci) [61] and 59 explicit SNP probes (‘rs’ probes) [62] were removed from the dataset. 814,341 probes remained after quality control and filtering. Loci showing differences in methylation (*β*) of > 0.2 between technical duplicates were removed to retain only those CpG sites showing high correlation of DNA methylation between duplicate samples. Differences in methylation (∆*β*) were calculated by subtracting the DNA methylation levels at each CpG site in the cells overexpressing each DNMT isoform from the corresponding DNA methylation of the control cell (‘Myc’).

### Treatment of DNMT-expressing cells with caffeic acid

Cells overexpressing DNMTs were cultured with 100 and 200 µM caffeic acid for 48 h at 37 °C in a 5% CO_2_ humidified atmosphere. These concentrations were determined based upon assessment of cell viability following exposure to 10–200 µM for 12, 24, 48 and 72 h (data not shown).

### Quantification of DNA methylation by bisulfite PCR pyrosequencing

Analysis of DNA methylation in response to treatment with caffeic acid was performed by pyrosequencing. DNA samples were bisulfite-converted using the EZ DNA methylation-Gold™ kit (Zymo research, USA) according to the manufacturer’s instructions. Global and locus-specific DNA methylation were quantified using PyroMark Q96 MD (Qiagen, USA). All primers were designed using MethPrimer [63].

### DNMT inhibitor assay

Cells overexpressing DNMTΔ3B4 were cultured in 100-mm dishes until reaching 80% confluence. Total protein was extracted using Mammalian protein extraction reagent (M-PER) (Thermo Fisher Scientific, UK). The exogenous DNMT protein tagged with c-Myc was isolated using Pierce c-Myc-Tag IP/Co-IP kit (Thermo Fisher Scientific, UK) according to the manufacturer’s instructions. Inhibition of DNMTΔ3B4 by 25–300 µM caffeic acid was quantified using the DNMT activity assay (Abcam, UK) according to the manufacturer’s instructions.

### Statistical analysis

Statistical analyses were performed using the IBM® SPSS statistical software program (version 24), GraphPad Prism (version 9.2.0) and R Studio (version 1.1.442). Data are presented as mean ± standard deviation (SD) from three independent experiments for qRT-PCR, pyrosequencing and DNMT inhibition assays. P-values were adjusted by the Benjamini–Hochberg method, with values of ≤ 0.05 considered statistically significant. Associations between methylation changes (∆β) and repetitive element evolutionary age were performed by linear regression, using estimates of subfamily age developed by Kapitonov et al. [64] and Khan et al. [65]. A two-sample Kolmogorov–Smirnov test was used to identify significant changes of DNA methylation, global methylation and DNMT inhibition of selected cells overexpressing DNMTs after treatment with dietary constituents.

## Supplementary Information

**Additional file 1. Supplementary Table 1**. Amino acid sequences of DNMT isoforms;** Supplementary Table 2**. Frequency of gains and losses of methylation by DNMT isoform overexpression;** Supplementary Table 3**. KEGG pathway analysis for loci hypermethylated in response to DNMT isoform overexpression;** Supplementary Table 4**. Primers used for analysis of exogenous DNMT isoform expression;** Supplementary Figure 1**. Endogenous expression of DNMT isoforms in HEK293T cells. Expression was assessed in control cells by qPCR. Due to high sequence similarity between some isoforms (DNMT3B1-5, DNMTD3B1/2, and DNMTD3B3/4), expression levels for individual isoforms could not be determined;** Supplementary Figure 2**. Gains of methylation in relation to CpG density. Frequency of gains of methylation (Db>0.2) at CpG sites mapping to CpG islands (green), shores (yellow), shelves (brown) and open seas (blue) by DNMT isoform;** Supplementary Figure 3**. Verification of target loci by pyrosequencing. Six loci identified as targets of DNMT1 (cg09559735), DNMT3A2 (cg02732111), DNMTD3B2 (cg25533247), DNMTD3B3 (cg08927738, cg12150401), DNMTD3B4 (cg22976313) and DNMT3L (cg08927738, cg12150401) were interrogated in DNA extracted from overexpressing and control (‘Myc’) cells. Beta values from the Illumina Infinium EPIC microarray were converted to percentages for comparison;** Supplementary Figure 4**. Density plot of genome-wide CpG methylation (black) and on chrX (pink).

## Data Availability

The datasets used and/or analysed during the current study are available from the corresponding author on reasonable request.
