# Supplementary material for: Genomic targets and selective inhibition of DNA methyltransferase isoforms
Source: Clin Epigenetics. 2022 Aug 20;14:103. doi: 10.1186/s13148-022-01325-4 (PMC9392947; doi:10.1186/s13148-022-01325-4)
Supplement: Supplementary file 1 — Additional file 1. Supplementary Table 1. Amino acid sequences of DNMT isoforms; Supplementary Table 2. Frequency of gains and losses of methylation by DNMT isoform overexpression; Supplementary Table 3. KEGG pathway analysis for loci hypermethylated in response to DNMT isoform overexpression; Supplementary Table 4. Primers used for analysis of exogenous DNMT isoform expression; Supplementary Figure 1. Endogenous expression of DNMT isoforms in HEK293T cells. Expression was assessed in control cells by qPCR. Due to high sequence similarity between some isoforms (DNMT3B1-5, DNMTD3B1/2, and DNMTD3B3/4), expression levels for individual isoforms could not be determined; Supplementary Figure 2. Gains of methylation in relation to CpG density. Frequency of gains of methylation (Db>0.2) at CpG sites mapping to CpG islands (green), shores (yellow), shelves (brown) and open seas (blue) by DNMT isoform; Supplementary Figure 3. Verification of target loci by pyrosequencing. Six loci identified as targets of DNMT1 (cg09559735), DNMT3A2 (cg02732111), DNMTD3B2 (cg25533247), DNMTD3B3 (cg08927738, cg12150401), DNMTD3B4 (cg22976313) and DNMT3L (cg08927738, cg12150401) were interrogated in DNA extracted from overexpressing and control (‘Myc’) cells. Beta values from the Illumina Infinium EPIC microarray were converted to percentages for comparison; Supplementary Figure 4. Density plot of genome-wide CpG methylation (black) and on chrX (pink). [file 13148_2022_1325_MOESM1_ESM.docx]

**Supplementary Table 1: Amino acid sequences of DNMT isoforms**

| **DNMT isoform** | **Amino acid sequence** |
| --- | --- |
| DNMT1 | MPARTAPARV PTLAVPAISL PDDVRRRLKD LERDSLTEKE CVKEKLNLLH EFLQTEIKNQ LCDLETKLRK EELSEEGYLA KVKSLLNKDL SLENGAHAYN REVNGRLENG NQARSEARRV GMADANSPPK PLSKPRTPRR SKSDGEAKPE PSPSPRITRK STRQTTITSH FAKGPAKRKP QEESERAKSD ESIKEEDKDQ DEKRRRVTSR ERVARPLPAE EPERAKSGTR TEKEEERDEK EEKRLRSQTK EPTPKQKLKE EPDREARAGV QADEDEDGDE KDEKKHRSQP KDLAAKRRPE EKEPEKVNPQ ISDEKDEDEK EEKRRKTTPK EPTEKKMARA KTVMNSKTHP  PKCIQCGQYL DDPDLKYGQH PPDAVDEPQM LTNEKLSIFD ANESGFESYE ALPQHKLTCF SVYCKHGHLC PIDTGLIEKN IELFFSGSAK PIYDDDPSLE GGVNGKNLGP INEWWITGFD GGEKALIGFS TSFAEYILMD PSPEYAPIFG LMQEKIYISK IVVEFLQSNS DSTYEDLINK IETTVPPSGL NLNRFTEDSL LRHAQFVVEQ VESYDEAGDS DEQPIFLTPC MRDLIKLAGV TLGQRRAQAR RQTIRHSTRE KDRGPTKATT TKLVYQIFDT FFAEQIEKDD REDKENAFKR RRCGVCEVCQ QPECGKCKAC KDMVKFGGSG RSKQACQERR CPNMAMKEAD  DDEEVDDNIP EMPSPKKMHQ GKKKKQNKNR ISWVGEAVKT DGKKSYYKKV CIDAETLEVG DCVSVIPDDS SKPLYLARVT ALWEDSSNGQ MFHAHWFCAG TDTVLGATSD PLELFLVDEC EDMQLSYIHS KVKVIYKAPS ENWAMEGGMD PESLLEGDDG KTYFYQLWYD QDYARFESPP KTQPTEDNKF KFCVSCARLA EMRQKEIPRV LEQLEDLDSR VLYYSATKNG ILYRVGDGVY LPPEAFTFNI KLSSPVKRPR KEPVDEDLYP EHYRKYSDYI KGSNLDAPEP YRIGRIKEIF CPKKSNGRPN ETDIKIRVNK FYRPENTHKS TPASYHADIN LLYWSDEEAV  VDFKAVQGRC TVEYGEDLPE CVQVYSMGGP NRFYFLEAYN AKSKSFEDPP NHARSPGNKG KGKGKGKGKP KSQACEPSEP EIEIKLPKLR TLDVFSGCGG LSEGFHQAGI SDTLWAIEMW DPAAQAFRLN NPGSTVFTED CNILLKLVMA GETTNSRGQR LPQKGDVEML CGGPPCQGFS GMNRFNSRTY SKFKNSLVVS FLSYCDYYRP RFFLLENVRN FVSFKRSMVL KLTLRCLVRM GYQCTFGVLQ AGQYGVAQTR RRAIILAAAP GEKLPLFPEP LHVFAPRACQ LSVVVDDKKF VSNITRLSSG PFRTITVRDT MSDLPEVRNG ASALEISYNG EPQSWFQRQL  RGAQYQPILR DHICKDMSAL VAARMRHIPL APGSDWRDLP NIEVRLSDGT MARKLRYTHH DRKNGRSSSG ALRGVCSCVE AGKACDPAAR QFNTLIPWCL PHTGNRHNHW AGLYGRLEWD GFFSTTVTNP EPMGKQGRVL HPEQHRVVSV RECARSQGFP DTYRLFGNIL DKHRQVGNAV PPPLAKAIGL EIKLCMLAKA RESASAKIKE EEAAKD |
| DNMT3A1 | MPAMPSSGPG DTSSSAAERE EDRKDGEEQE EPRGKEERQE PSTTARKVGR PGRKRKHPPV ESGDTPKDPA VISKSPSMAQ DSGASELLPN GDLEKRSEPQ PEEGSPAGGQ KGGAPAEGEG AAETLPEASR AVENGCCTPK EGRGAPAEAG KEQKETNIES MKMEGSRGRL RGGLGWESSL RQRPMPRLTF QAGDPYYISK RKRDEWLARW KREAEKKAKV IAGMNAVEEN QGPGESQKVE EASPPAVQQP TDPASPTVAT TPEPVGSDAG DKNATKAGDD EPEYEDGRGF GIGELVWGKL RGFSWWPGRI VSWWMTGRSR AAEGTRWVMW FGDGKFSVVC VEKLMPLSSF  CSAFHQATYN KQPMYRKAIY EVLQVASSRA GKLFPVCHDS DESDTAKAVE VQNKPMIEWA LGGFQPSGPK GLEPPEEEKN PYKEVYTDMW VEPEAAAYAP PPPAKKPRKS TAEKPKVKEI IDERTRERLV YEVRQKCRNI EDICISCGSL NVTLEHPLFV GGMCQNCKNC FLECAYQYDD DGYQSYCTIC CGGREVLMCG NNNCCRCFCV ECVDLLVGPG AAQAAIKEDP WNCYMCGHKG TYGLLRRRED WPSRLQMFFA NNHDQEFDPP KVYPPVPAEK RKPIRVLSLF DGIATGLLVL KDLGIQVDRY IASEVCEDSI TVGMVRHQGK IMYVGDVRSV TQKHIQEWGP  FDLVIGGSPC NDLSIVNPAR KGLYEGTGRL FFEFYRLLHD ARPKEGDDRP FFWLFENVVA MGVSDKRDIS RFLESNPVMI DAKEVSAAHR ARYFWGNLPG MNRPLASTVN DKLELQECLE HGRIAKFSKV RTITTRSNSI KQGKDQHFPV FMNEKEDILW CTEMERVFGF PVHYTDVSNM SRLARQRLLG RSWSVPVIRH LFAPLKEYFA  CV |
| DNMT3A2 | MNAVEENQGP GESQKVEEAS PPAVQQPTDP ASPTVATTPE PVGSDAGDKN ATKAGDDEPE YEDGRGFGIG ELVWGKLRGF SWWPGRIVSW WMTGRSRAAE GTRWVMWFGD GKFSVVCVEK LMPLSSFCSA FHQATYNKQP MYRKAIYEVL QVASSRAGKL FPVCHDSDES DTAKAVEVQN KPMIEWALGG FQPSGPKGLE PPEEEKNPYK EVYTDMWVEP EAAAYAPPPP AKKPRKSTAE KPKVKEIIDE RTRERLVYEV RQKCRNIEDI CISCGSLNVT LEHPLFVGGM CQNCKNCFLE CAYQYDDDGY QSYCTICCGG REVLMCGNNN CCRCFCVECV DLLVGPGAAQ  AAIKEDPWNC YMCGHKGTYG LLRRREDWPS RLQMFFANNH DQEFDPPKVY PPVPAEKRKP IRVLSLFDGI ATGLLVLKDL GIQVDRYIAS EVCEDSITVG MVRHQGKIMY VGDVRSVTQK HIQEWGPFDL VIGGSPCNDL SIVNPARKGL YEGTGRLFFE FYRLLHDARP KEGDDRPFFW LFENVVAMGV SDKRDISRFL ESNPVMIDAK EVSAAHRARY FWGNLPGMNR PLASTVNDKL ELQECLEHGR IAKFSKVRTI TTRSNSIKQG KDQHFPVFMN EKEDILWCTE MERVFGFPVH YTDVSNMSRL ARQRLLGRSW SVPVIRHLFA PLKEYFACV |
| DNMT3B1 | MKGDTRHLNG EEDAGGREDS ILVNGACSDQ SSDSPPILEA IRTPEIRGRR SSSRLSKREV SSLLSYTQDL TGDGDGEDGD GSDTPVMPKL FRETRTRSES PAVRTRNNNS VSSRERHRPS PRSTRGRQGR NHVDESPVEF PATRSLRRRA TASAGTPWPS PPSSYLTIDL TDDTEDTHGT PQSSSTPYAR LAQDSQQGGM ESPQVEADSG DGDSSEYQDG KEFGIGDLVW GKIKGFSWWP AMVVSWKATS KRQAMSGMRW VQWFGDGKFS EVSADKLVAL GLFSQHFNLA TFNKLVSYRK AMYHALEKAR VRAGKTFPSS PGDSLEDQLK PMLEWAHGGF KPTGIEGLKP  NNTQPVVNKS KVRRAGSRKL ESRKYENKTR RRTADDSATS DYCPAPKRLK TNCYNNGKDR GDEDQSREQM ASDVANNKSS LEDGCLSCGR KNPVSFHPLF EGGLCQTCRD RFLELFYMYD DDGYQSYCTV CCEGRELLLC SNTSCCRCFC VECLEVLVGT GTAAEAKLQE PWSCYMCLPQ RCHGVLRRRK DWNVRLQAFF TSDTGLEYEA PKLYPAIPAA RRRPIRVLSL FDGIATGYLV LKELGIKVGK YVASEVCEES IAVGTVKHEG NIKYVNDVRN ITKKNIEEWG PFDLVIGGSP CNDLSNVNPA RKGLYEGTGR LFFEFYHLLN YSRPKEGDDR PFFWMFENVV  AMKVGDKRDI SRFLECNPVM IDAIKVSAAH RARYFWGNLP GMNRPVIASK NDKLELQDCL EYNRIAKLKK VQTITTKSNS IKQGKNQLFP VVMNGKEDVL WCTELERIFG FPVHYTDVSN MGRGARQKLL GRSWSVPVIR HLFAPLKDYF ACE |
| DNMT3B2 | MKGDTRHLNG EEDAGGREDS ILVNGACSDQ SSDSPPILEA IRTPEIRGRR SSSRLSKREV SSLLSYTQDL TGDGDGEDGD GSDTPVMPKL FRETRTRSES PAVRTRNNNS VSSRERHRPS PRSTRGRQGR NHVDESPVEF PATRSLRRRA TASAGTPWPS PPSSYLTIDL TDDTEDTHGT PQSSSTPYAR LAQDSQQGGM ESPQVEADSG DGDSSEYQDG KEFGIGDLVW GKIKGFSWWP AMVVSWKATS KRQAMSGMRW VQWFGDGKFS EVSADKLVAL GLFSQHFNLA TFNKLVSYRK AMYHALEKAR VRAGKTFPSS PGDSLEDQLK PMLEWAHGGF KPTGIEGLKP  NNTQPENKTR RRTADDSATS DYCPAPKRLK TNCYNNGKDR GDEDQSREQM ASDVANNKSS LEDGCLSCGR KNPVSFHPLF EGGLCQTCRD RFLELFYMYD DDGYQSYCTV CCEGRELLLC SNTSCCRCFC VECLEVLVGT GTAAEAKLQE PWSCYMCLPQ RCHGVLRRRK DWNVRLQAFF TSDTGLEYEA PKLYPAIPAA RRRPIRVLSL FDGIATGYLV LKELGIKVGK YVASEVCEES IAVGTVKHEG NIKYVNDVRN ITKKNIEEWG PFDLVIGGSP CNDLSNVNPA RKGLYEGTGR LFFEFYHLLN YSRPKEGDDR PFFWMFENVV AMKVGDKRDI SRFLECNPVM  IDAIKVSAAH RARYFWGNLP GMNRPVIASK NDKLELQDCL EYNRIAKLKK VQTITTKSNS IKQGKNQLFP VVMNGKEDVL WCTELERIFG FPVHYTDVSN MGRGARQKLL GRSWSVPVIR HLFAPLKDYF ACE |
| DNMT3B3 | MKGDTRHLNG EEDAGGREDS ILVNGACSDQ SSDSPPILEA IRTPEIRGRR SSSRLSKREV SSLLSYTQDL TGDGDGEDGD GSDTPVMPKL FRETRTRSES PAVRTRNNNS VSSRERHRPS PRSTRGRQGR NHVDESPVEF PATRSLRRRA TASAGTPWPS PPSSYLTIDL TDDTEDTHGT PQSSSTPYAR LAQDSQQGGM ESPQVEADSG DGDSSEYQDG KEFGIGDLVW GKIKGFSWWP AMVVSWKATS KRQAMSGMRW VQWFGDGKFS EVSADKLVAL GLFSQHFNLA TFNKLVSYRK AMYHALEKAR VRAGKTFPSS PGDSLEDQLK PMLEWAHGGF KPTGIEGLKP NNTQPENKTR RRTADDSATS DYCPAPKRLK TNCYNNGKDR GDEDQSREQM ASDVANNKSS LEDGCLSCGR KNPVSFHPLF EGGLCQTCRD RFLELFYMYD DDGYQSYCTV CCEGRELLLC SNTSCCRCFC VECLEVLVGT GTAAEAKLQE PWSCYMCLPQ RCHGVLRRRK DWNVRLQAFF TSDTGLEYEA PKLYPAIPAA RRRPIRVLSL FDGIATGYLV LKELGIKVGK YVASEVCEES IAVGTVKHEG NIKYVNDVRN ITKKNIEEWG PFDLVIGGSP CNDLSNVNPA RKGLYEGTGR LFFEFYHLLN YSRPKEGDDR PFFWMFENVV AMKVGDKRDI SRFLECNPVM  IDAIKVSAAH RARYFWGNLP GMNRIFGFPV HYTDVSNMGR GARQKLLGRS WSVPVIRHLF APLKDYFACE |
| DNMT3B4 | MKGDTRHLNG EEDAGGREDS ILVNGACSDQ SSDSPPILEA IRTPEIRGRR SSSRLSKREV SSLLSYTQDL TGDGDGEDGD GSDTPVMPKL FRETRTRSES PAVRTRNNNS VSSRERHRPS PRSTRGRQGR NHVDESPVEF PATRSLRRRA TASAGTPWPS PPSSYLTIDL TDDTEDTHGT PQSSSTPYAR LAQDSQQGGM ESPQVEADSG DGDSSEYQDG KEFGIGDLVW GKIKGFSWWP AMVVSWKATS KRQAMSGMRW VQWFGDGKFS EVSADKLVAL GLFSQHFNLA TFNKLVSYRK AMYHALEKAR VRAGKTFPSS PGDSLEDQLK PMLEWAHGGF KPTGIEGLKP  NNTQPENKTR RRTADDSATS DYCPAPKRLK TNCYNNGKDR GDEDQSREQM ASDVANNKSS LEDGCLSCGR KNPVSFHPLF EGGLCQTCRD RFLELFYMYD DDGYQSYCTV CCEGRELLLC SNTSCCRCFC VECLEVLVGT GTAAEAKLQE PWSCYMCLPQ RCHGVLRRRK DWNVRLQAFF TSDTGLEYEA PKLYPAIPAA RRRPIRVLSL FDGIATGYLV LKELGIKVGK YVASEVCEES IAVGTVKHEG NIKYVNDVRN ITKKNIEEWG PFDLVIGGSP CNDLSNVNPA RKGLYEGTGR LFFEFYHLLN YSRPKEGDDR PFFWMFENVV AMKVGDKRDI SRFLECNPVM  IDAIKVSAAH RARYFWGNLP GMNS |
| DNMT3B5 | MKGDTRHLNG EEDAGGREDS ILVNGACSDQ SSDSPPILEA IRTPEIRGRR SSSRLSKREV SSLLSYTQDL TGDGDGEDGD GSDTPVMPKL FRETRTRSES PAVRTRNNNS VSSRERHRPS PRSTRGRQGR NHVDESPVEF PATRSLRRRA TASAGTPWPS PPSSYLTIDL TDDTEDTHGT PQSSSTPYAR LAQDSQQGGM ESPQVEADSG DGDSSEYQDG KEFGIGDLVW GKIKGFSWWP AMVVSWKATS KRQAMSGMRW VQWFGDGKFS EVSADKLVAL GLFSQHFNLA TFNKLVSYRK AMYHALEKAR VRAGKTFPSS PGDSLEDQLK PMLEWAHGGF KPTGIEGLKP  NNTQPENKTR RRTADDSATS DYCPAPKRLK TNCYNNGKDR GDEDQSREQM ASDVANNKSS LEDGCLSCGR KNPVSFHPLF EGGLCQTCRD RFLELFYMYD DDGYQSYCTV CCEGRELLLC SNTSCCRCFC VECLEVLVGT GTAAEAKLQE PWSCYMCLPQ RCHGVLRRRK DWNVRLQAFF TSDTGLEYEA PKLYPAIPAA RRRPIRVLSL FDGIATGYLV LKELGIKVGK YVASEVCEES IAVGTVKHEG NIKYVNDVRN ITKKNIEEWG PFDLVIGGSP CNDLSNVNPA RKGLYEGTGR LFFEFYHLLN YSRPKEGDDR PFFWMFENVV AMKVGDKRDI SRFLECNPVM  IDAIKVSAAH RARYFWGNLP GMNRPVIASK NDKLELQDCL EYNRIARIFG FPVHYTDVSN MGRGARQKLL GRSWSVPVIR HLFAPLKDYF ACE |
| DNMTΔ3B1 | MESPQVEADS GDGDSSEYQD GKEFGIGDLV WGKIKGFSWW PAMVVSWKAT SKRQAMSGMR WVQWFGDGKF SEVSADKLVA LGLFSQHFNL ATFNKLVSYR KAMYHALEKA RVRAGKTFPS SPGDSLEDQL KPMLEWAHGG FKPTGIEGLK PNNTQPVVNK SKVRRAGSRK LESRKYENKT RRRTADDSAT SDYCPAPKRL KTNCYNNGKD RGDEDQSREQ MASDVANNKS SLEDGCLSCG RKNPVSFHPL FEGGLCQTCR DRFLELFYMY DDDGYQSYCT VCCEGRELLL CSNTSCCRCF CVECLEVLVG TGTAAEAKLQ EPWSCYMCLP QRCHGVLRRR KDWNVRLQAF  FTSDTGLEYE APKLYPAIPA ARRRPIRVLS LFDGIATGYL VLKELGIKVG KYVASEVCEE SIAVGTVKHE GNIKYVNDVR NITKKNIEEW GPFDLVIGGS PCNDLSNVNP ARKGLYEGTG RLFFEFYHLL NYSRPKEGDD RPFFWMFENV VAMKVGDKRD ISRFLECNPV MIDAIKVSAA HRARYFWGNL PGMNRPVIAS KNDKLELQDC LEYNRIAKLK KVQTITTKSN SIKQGKNQLF PVVMNGKEDV LWCTELERIF GFPVHYTDVS NMGRGARQKL LGRSWSVPVI RHLFAPLKDY FACE |
| DNMTΔ3B2 | MESPQVEADS GDGDSSEYQD GKEFGIGDLV WGKIKGFSWW PAMVVSWKAT SKRQAMSGMR WVQWFGDGKF SEVSADKLVA LGLFSQHFNL ATFNKLVSYR KAMYHALEKA RVRAGKTFPS SPGDSLEDQL KPMLEWAHGG FKPTGIEGLK PNNTQPENKT RRRTADDSAT SDYCPAPKRL KTNCYNNGKD RGDEDQSREQ MASDVANNKS SLEDGCLSCG RKNPVSFHPL FEGGLCQTCR DRFLELFYMY DDDGYQSYCT VCCEGRELLL CSNTSCCRCF CVECLEVLVG TGTAAEAKLQ EPWSCYMCLP QRCHGVLRRR KDWNVRLQAF FTSDTGLEYE APKLYPAIPA  ARRRPIRVLS LFDGIATGYL VLKELGIKVG KYVASEVCEE SIAVGTVKHE GNIKYVNDVR NITKKNIEEW GPFDLVIGGS PCNDLSNVNP ARKGLYEGTG RLFFEFYHLL NYSRPKEGDD RPFFWMFENV VAMKVGDKRD ISRFLECNPV MIDAIKVSAA HRARYFWGNL PGMNRPVIAS KNDKLELQDC LEYNRIAKLK KVQTITTKSN SIKQGKNQLF PVVMNGKEDV LWCTELERIF GFPVHYTDVS NMGRGARQKL LGRSWSVPVI RHLFAPLKDY FACE |
| DNMTΔ3B3 | MESPQVEADS GDGDSSEYQV SADKLVALGL FSQHFNLATF NKLVSYRKAM YHALEKARVR AGKTFPSSPG DSLEDQLKPM LEWAHGGFKP TGIEGLKPNN TQPVVNKSKV RRAGSRKLES RKYENKTRRR TADDSATSDY CPAPKRLKTN CYNNGKDRGD EDQSREQMAS DVANNKSSLE DGCLSCGRKN PVSFHPLFEG GLCQTCRDRF LELFYMYDDD GYQSYCTVCC EGRELLLCSN TSCCRCFCVE CLEVLVGTGT AAEAKLQEPW SCYMCLPQRC HGVLRRRKDW NVRLQAFFTS DTGLEYEAPK LYPAIPAARR RPIRVLSLFD GIATGYLVLK ELGIKVGKYV  ASEVCEESIA VGTVKHEGNI KYVNDVRNIT KKNIEEWGPF DLVIGGSPCN DLSNVNPARK GLYEGTGRLF FEFYHLLNYS RPKEGDDRPF FWMFENVVAM KVGDKRDISR FLECNPVMID AIKVSAAHRA RYFWGNLPGM NRPVIASKND KLELQDCLEY NRIAKLKKVQ TITTKSNSIK QGKNQLFPVV MNGKEDVLWC TELERIFGFP VHYTDVSNMG RGARQKLLGR SWSVPVIRHL FAPLKDYFAC E |
| DNMTΔ3B4 | MESPQVEADS GDGDSSEYQV SADKLVALGL FSQHFNLATF NKLVSYRKAM YHALEKARVR AGKTFPSSPG DSLEDQLKPM LEWAHGGFKP TGIEGLKPNN TQPENKTRRR TADDSATSDY CPAPKRLKTN CYNNGKDRGD EDQSREQMAS DVANNKSSLE DGCLSCGRKN PVSFHPLFEG GLCQTCRDRF LELFYMYDDD GYQSYCTVCC EGRELLLCSN TSCCRCFCVE CLEVLVGTGT AAEAKLQEPW SCYMCLPQRC HGVLRRRKDW NVRLQAFFTS DTGLEYEAPK LYPAIPAARR RPIRVLSLFD GIATGYLVLK ELGIKVGKYV ASEVCEESIA VGTVKHEGNI  KYVNDVRNIT KKNIEEWGPF DLVIGGSPCN DLSNVNPARK GLYEGTGRLF FEFYHLLNYS RPKEGDDRPF FWMFENVVAM KVGDKRDISR FLECNPVMID AIKVSAAHRA RYFWGNLPGM NRPVIASKND KLELQDCLEY NRIAKLKKVQ TITTKSNSIK QGKNQLFPVV MNGKEDVLWC TELERIFGFP VHYTDVSNMG RGARQKLLGR SWSVPVIRHL FAPLKDYFAC E |
| DNMT3L | MAAIPALDPE AEPSMDVILV GSSELSSSVS PGTGRDLIAY EVKANQRNIE DICICCGSLQ VHTQHPLFEG GICAPCKDKF LDALFLYDDD GYQSYCSICC SGETLLICGN PDCTRCYCFE CVDSLVGPGT SGKVHAMSNW VCYLCLPSSR SGLLQRRRKW RSQLKAFYDR ESENPLEMFE TVPVWRRQPV RVLSLFEDIK KELTSLGFLE SGSDPGQLKH VVDVTDTVRK DVEEWGPFDL VYGATPPLGH TCDRPPSWYL FQFHRLLQYA RPKPGSPRPF FWMFVDNLVL NKEDLDVASR FLEMEPVTIP DVHGGSLQNA VRVWSNIPAI RSRHWALVSE EELSLLAQNK  QSSKLAAKWP TKLVKNCFLP LREYFKYFST ELTSSL |

**Supplementary Table 2: Frequency of gains and losses of methylation by DNMT isoform overexpression**

|  | Loss of methylation | | | | Gains of methylation | | | |
| --- | --- | --- | --- | --- | --- | --- | --- | --- |
|  | Δβ < -0.4 | Δβ < -0.3 | Δβ < -0.2 | Δβ < -0.1 | Δβ > 0.1 | Δβ > 0.2 | Δβ > 0.3 | Δβ > 0.4 |
| DNMT1 | 103 | 526 | 2716 | 15732 | 24871 | 5631 | 1306 | 267 |
| DNMT3A1 | 131 | 721 | 3779 | 20921 | 19330 | 3919 | 820 | 147 |
| DNMT3A2 | 113 | 614 | 2964 | 17903 | 46964 | 16115 | 5008 | 1207 |
| DNMT3B1 | 30 | 220 | 1415 | 10350 | 34794 | 7288 | 1502 | 256 |
| DNMT3B2 | 84 | 485 | 2915 | 20271 | 25181 | 4992 | 1031 | 198 |
| DNMT3B3 | 219 | 1226 | 5759 | 27654 | 13649 | 2226 | 358 | 54 |
| DNMT3B4 | 230 | 1191 | 4808 | 22024 | 33054 | 8655 | 2163 | 467 |
| DNMT3B5 | 152 | 919 | 4311 | 20935 | 17955 | 3051 | 642 | 122 |
| DNMTΔ3B1 | 76 | 386 | 2253 | 12253 | 31409 | 6808 | 1494 | 269 |
| DNMTΔ3B2 | 9 | 61 | 435 | 3520 | 47077 | 13581 | 3877 | 975 |
| DNMTΔ3B3 | 191 | 965 | 4391 | 23199 | 37504 | 9955 | 2610 | 568 |
| DNMTΔ3B4 | 24 | 187 | 1723 | 23996 | 52709 | 14150 | 3666 | 765 |
| DNMT3L | 13 | 156 | 1125 | 16539 | 48157 | 12104 | 2785 | 516 |

**Supplementary Table 3: KEGG pathway analysis for loci hypermethylated in response to DNMT isoform overexpression**

|  | DNMT1 | DNMT3L | DNMT3A1 | DNMT3A2 | DNMT3B1 | DNMT3B2 | DNMT3B3 | DNMT3B4 | DNMT3B5 | DNMTΔ3B1 | DNMTΔ3B2 | DNMTΔ3B3 | DNMTΔ3B4 |
| --- | --- | --- | --- | --- | --- | --- | --- | --- | --- | --- | --- | --- | --- |
| Nicotine addiction |  |  |  |  |  |  |  |  |  |  |  |  |  |
| Viral protein interaction with cytokine and cytokine receptor |  |  |  |  |  |  |  |  |  |  |  |  |  |
| Cytokine-cytokine receptor interaction |  |  |  |  |  |  |  |  |  |  |  |  |  |
| Mineral absorption |  |  |  |  |  |  |  |  |  |  |  |  |  |
| Neuroactive ligand-receptor interaction |  |  |  |  |  |  |  |  |  |  |  |  |  |
| Rap1 signalling pathway |  |  |  |  |  |  |  |  |  |  |  |  |  |
| Th17 cell differentiation |  |  |  |  |  |  |  |  |  |  |  |  |  |

**Supplementary Table 4: Primers used for analysis of exogenous DNMT isoform expression**

| **Target(s)** | **Primer sequence** |
| --- | --- |
| Myc | Forward: 5’ AGAAGCTGATCTCAGAGGAG 3’ |
| DNMT1 | Reverse: 5’ ATCGTCGGGCAGCGAGAT 3’ |
| DNMT3A1 | Reverse: 5’ CGCTCCGCAGCAGAGCT 3’ |
| DNMT3A2 | Reverse: 5’ ATCGTCGGGCAGCGAGAT 3’ |
| DNMT3B1-5 | Reverse: 5’ GTTGACGAGGATCGAGTCTT 3’ |
| DNMTΔ3B1-2 | Reverse: 5’ CTTCCCATCCTGATACTCTG 3’ |
| DNMTΔ3B3-4 | Reverse: 5’ TGCAGAGACCTGATACTCTG 3’ |
| DNMT3L | Reverse: 5’ CACTGGATCCCACCAAAATC 3’ |

**Supplementary Figure 1: Endogenous expression of DNMT isoforms in HEK293T cells.** Expression was assessed in control cells by qPCR. Due to high sequence similarity between some isoforms (DNMT3B1-5, DNMTΔ3B1/2, and DNMTΔ3B3/4), expression levels for individual isoforms could not be determined.

**
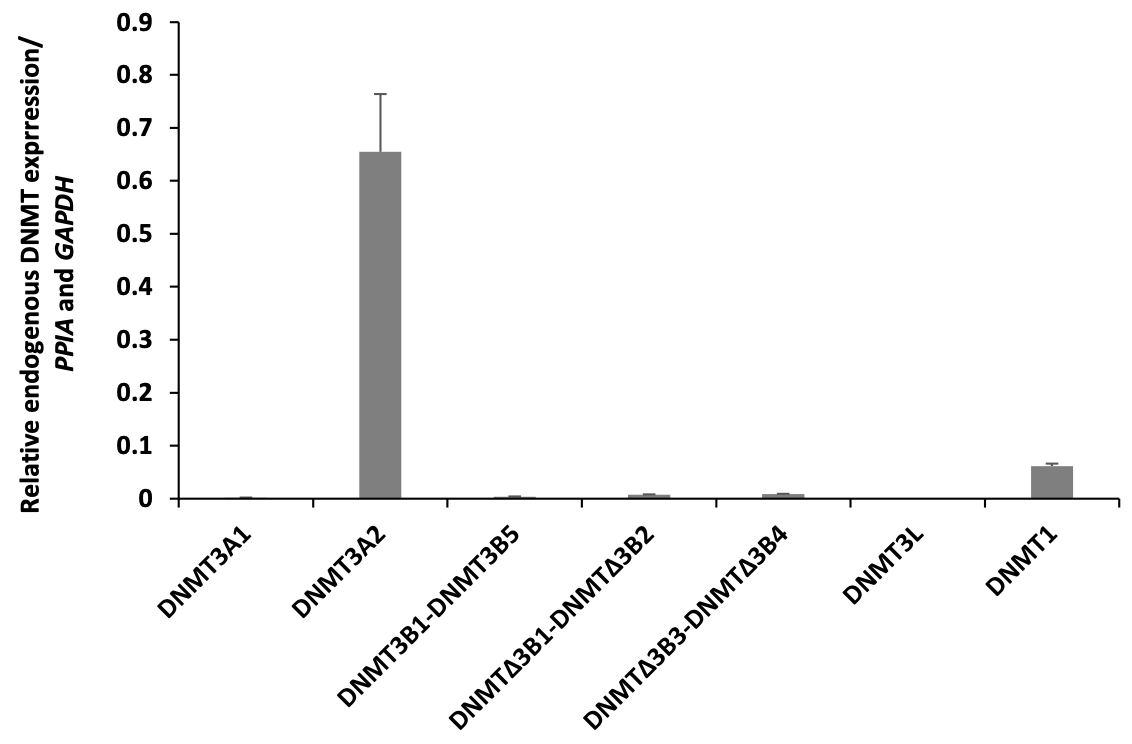
**

**Supplementary Figure 2: Gains of methylation in relation to CpG density.** Frequency of gains of methylation (Δβ>0.2) at CpG sites mapping to CpG islands (green), shores (yellow), shelves (brown) and open seas (blue) by DNMT isoform.

**
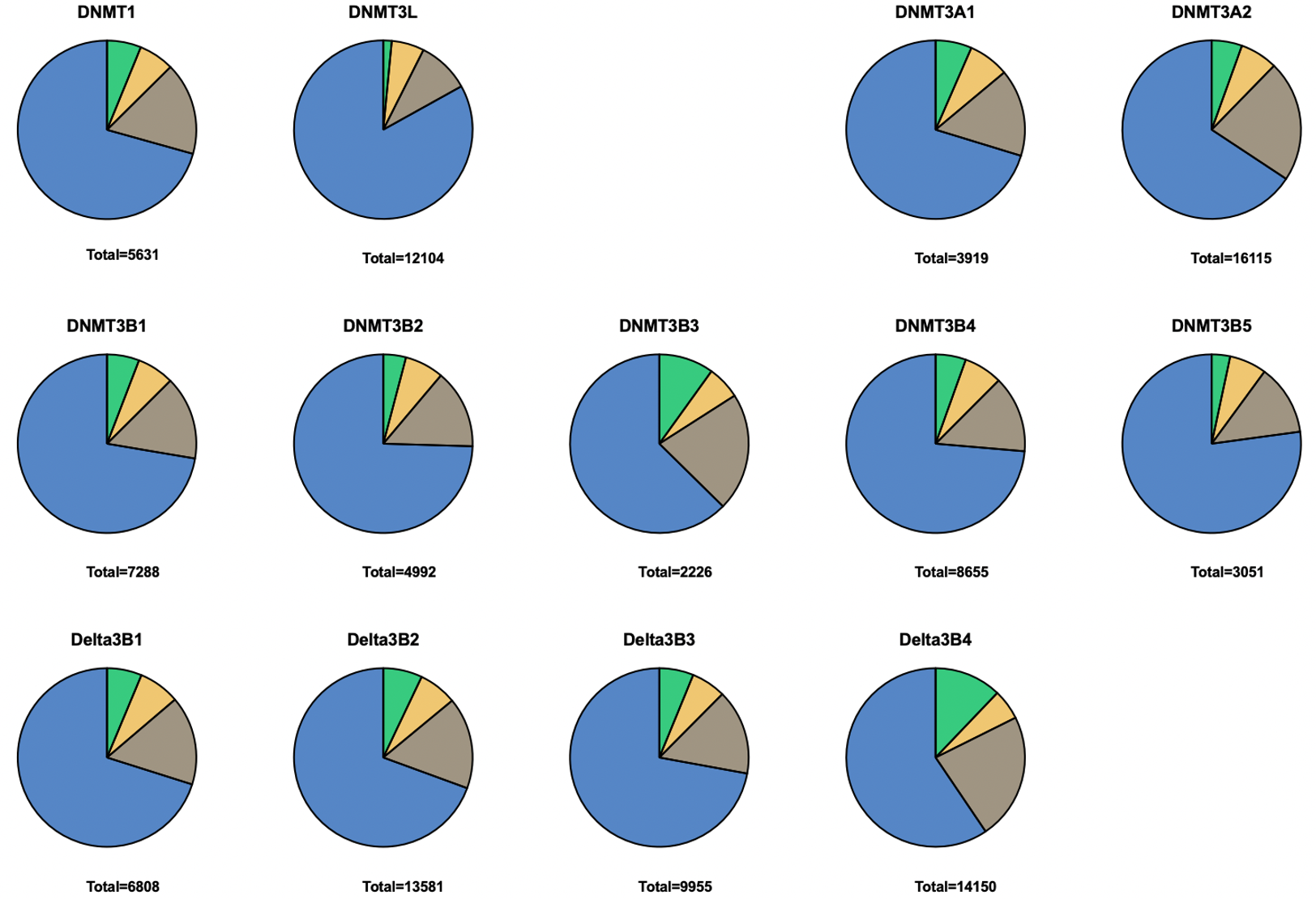
**

**Supplementary Figure 3: Verification of target loci by pyrosequencing.** Six loci identified as targets of DNMT1 (cg09559735), DNMT3A2 (cg02732111), DNMTΔ3B2 (cg25533247), DNMTΔ3B3 (cg08927738, cg12150401), DNMTΔ3B4 (cg22976313) and DNMT3L (cg08927738, cg12150401) were interrogated in DNA extracted from overexpressing and control (‘Myc’) cells. Beta values from the Illumina Infinium EPIC microarray were converted to percentages for comparison.

**
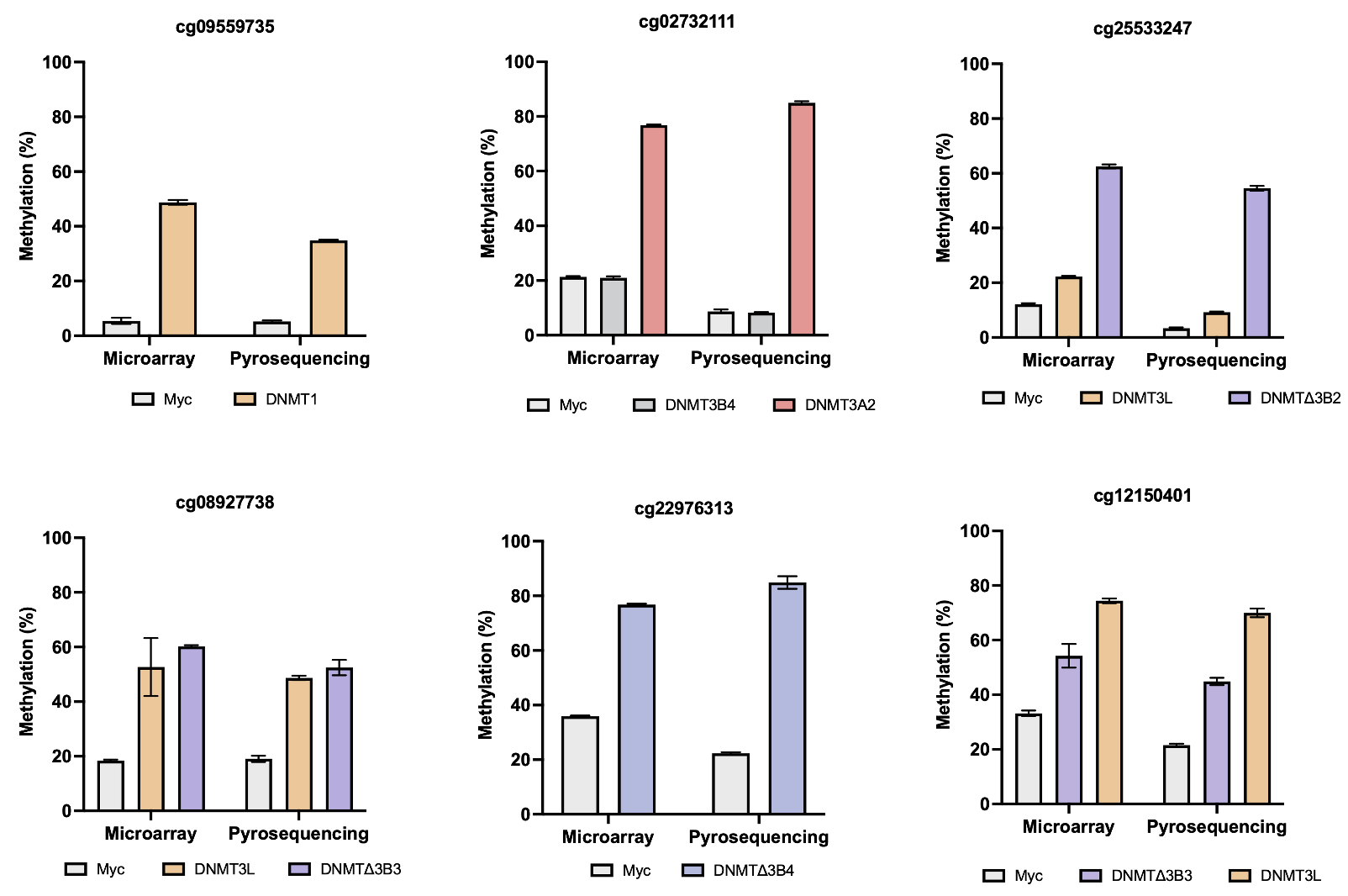
**

**Supplementary Figure 4: Density plot of genome-wide CpG methylation (black) and on chrX (pink)**

**
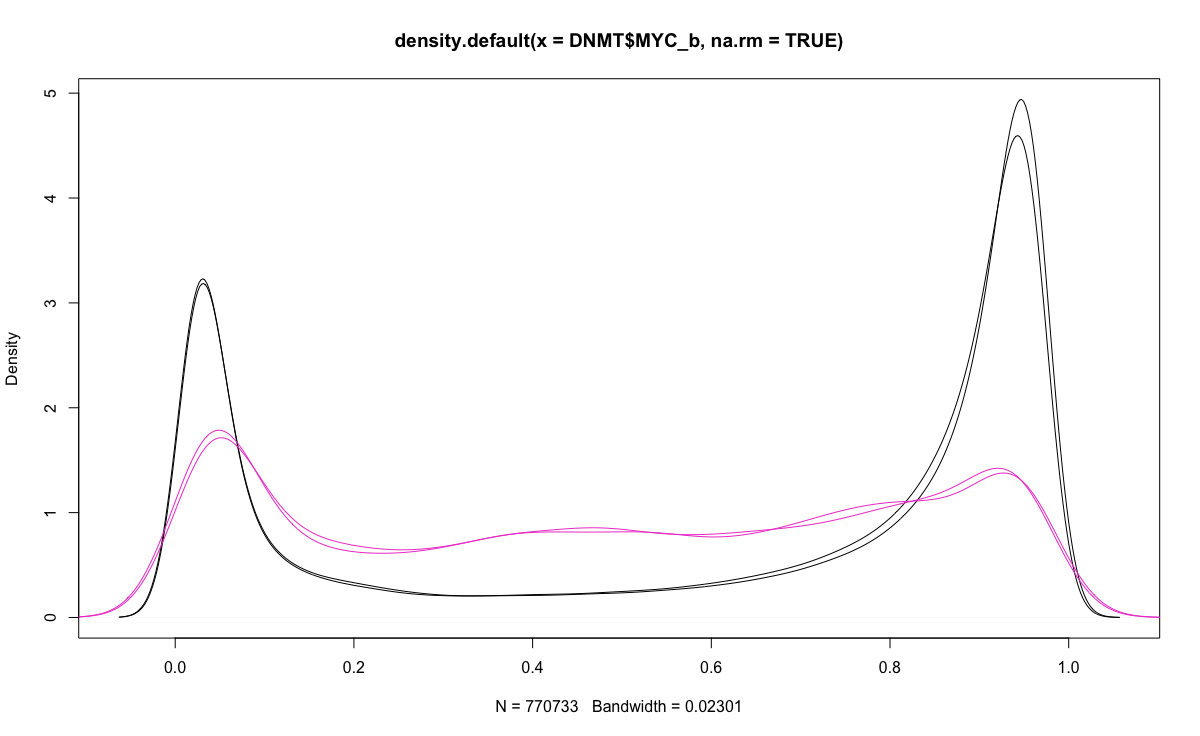
**
